# Study of wrap mode impact on *Pseudomonas aeruginosa* motion in the chemotactic field of fungi

**DOI:** 10.1038/s41540-026-00789-x

**Published:** 2026-07-20

**Authors:** Austin Hansen, Alireza Ramezani, Dale A. Pelletier, Tomás A. Rush, Roya Zandi, Yi-Syuan Guo, Huijing Du, William Cannon, Mark Alber

**Affiliations:** 1 Department of Mathematics, University of California, Riverside, CA USA; 2 Interdisciplinary Center for Data-driven Modeling in Biology, University of California, Riverside, CA USA; 3 Department of Physics and Astronomy, University of California, Riverside, CA USA; 4 Biosciences Division, Oak Ridge National Laboratory, Oak Ridge, TN USA; 5 Center for Nanophase Materials Sciences, Oak Ridge National Laboratory, Oak Ridge, TN USA; 6 Environmental Molecular Sciences Laboratory, Pacific Northwest National Laboratory, Richland, WA USA; 7 Department of Mathematics, University of Nebraska-Lincoln, Lincoln, NE USA; 8 Physical & Computational Sciences Directorate, Pacific Northwest National Laboratory, Richland, WA USA; 9 Mathematical Institute, Leiden University, Leiden, The Netherlands

**Keywords:** Computational biology and bioinformatics, Mathematics and computing, Microbiology

## Abstract

An agent-based discrete computational model biologically calibrated to *Pseudomonas aeruginosa* migration is used to explore the impacts of bacterial reversals and wrap mode on the efficiency of motion in different environments, both with and without chemotaxis. It is first shown that wrap mode increases the exploration of continuous multimodal chemotactic profiles such as those produced by biologically relevant fungal networks. For cells undergoing a run-reverse pattern, it is shown that the bacteria are likely to remain at the first local chemoattractant maximal production site on a hypha they find. However, with wrap mode, the bacteria can more easily escape these local sites to further explore their neighboring environment along the fungi, suggesting that wrap mode may be beneficial for migration along the fungi in liquid. In a different set of simulations of bacterial motion close to an isolated chemotactic source, wrap mode is shown to increase the ability of a bacterium to reorient toward the source while reducing the overall motion required for similar chemotactic efficiency as a run-reverse strategy, suggesting a potential metabolic benefit. In contrast, model simulations show that wrap mode can increase the rate of dispersal of *P. aeruginosa* in a nonchemotactic environment.

## Introduction

Bacterial motility is a widely studied field of microbiology that intersects many other disciplines, such as bioremediation, pathogenesis, and agriculture. In many of these fields, both the long-term distributions of the bacterial population and individual bacterial trajectories provide crucial information on the dynamics of the colony and its subsequent interactions with the surrounding environment^1–5^. For example, a recent study showed that of 23 different strains of *Pseudomonas*, several strains promoted root colonization by mycorrhizal fungi of 3 different Populus species^6^. However, for bacteria to be present in adequate quantities, chemotaxis is likely to play an important role by promoting bacterial colonization of the fungal network^7,8^. When near the fungi, bacteria can also disperse along hyphae and transfer beneficial metabolites to the tips of hyphae^9^.

Although there is a wide range of motility strategies in different bacteria, many bacteria use reversals in their migration to optimize their collective and individual motion. For example, it was shown that an optimal rate of reversals in their gliding direction is essential for efficient swarming of *Myxococcus xanthus*^10^. Furthermore, the maximum rate of protein spread in the colony is observed for cells that optimally reverse directions for swarming. (See, among others,^10–12^). In addition to gliding, flagellated motion is another common form of bacterial motility that can lead to different swimming patterns, depending on the number of flagella and their location along the cell body. For a review of the mechanisms of bacterial motility see ref. ^4^.

Both *Pseudomonas aeruginosa* and *Pseudomonas putida* have a single flagellum on one pole of their cell body and they move with three distinct modes of migration, namely push, pull and wrap mode where push-pull and pull-push transitions are separated by reversal events^13,14^. In push and pull modes, a bacterium swims approximately in a straight line, either pushed or pulled along by its flagellum.^13,14^. However, before some reversal events, a bacterium may enter what is known as wrap mode, in which the bacterium slows down and exhibits a prolonged change in orientation. In particular for *P. aeruginosa*, it was found in ref. ^14^ that 90% of the angle changes during reversals were between 150^∘^ and 180^∘^. In wrap mode the distribution of wrap mode was found to be from 0^∘^ to 180^∘^ with a peak of around 95^∘^. This leads bacteria to slow down and have a wider angular change when wrap mode occurs before a reversal as opposed to reversals preceded by only push or pull mode.

Wrap mode has been observed within a number of polarly flagellated bacteria^14–18^ however, its function and purpose are still debated and potentially vary between bacterial species. For bipolar flagellated bacteria, wrap mode seems to be important for both efficient motion and reorientation of the cell body. During runs, the front flagellum wraps around the cell body while the rear flagellum is extended behind, allowing for greater swimming speeds. In contrast, having both flagella wrap around the cell body appears to aid in reorientation of the cell body^19–21^. In *Shewanella putrefaciens* and *Burkholderia sp. RPE64*, studies have observed a different function. Here, wrap mode seems critically linked to a bacterium’s ability to escape from crevices in structured environments by allowing the bacterium to move backwards and free itself in a method named screw thread motility^17,18,22^. For mono-polar bacteria, such as *P. putida* and *P. aeruginosa*, wrap mode slows the bacteria, resulting in two distinct movement speeds and it is suggested that it improves aggregation/dispersal^13,14,16^. Several attempts to understand whether the wrap mode serves a specific biological function have utilized computational modeling, including models of the flagellum^23–25^, as well as agent-based and active particle models that predict long-term bacterial dynamics with and without chemotaxis^13,14,16^. Despite these studies, it is not fully understood whether the benefit of the wrap mode is largely due to the increased time before reversal events or increased angular exploration. Moreover, how wrap mode affects bacterial migration in multimodal chemotactic environments has largely been unstudied but is critical to understanding how bacteria may respond and aggregate around fungal networks.

To answer some of these questions, in this article we apply a general coarse-grained particle (Subcellular Elements (SCE)) approach that allows for bending and contraction of the bacterial cell, which was previously used for describing *P. aeruginosa* migration without chemotaxis^11,26–28^. Bending and contraction have been demonstrated to be an important aspect of bacterial dynamics near surfaces and in crowded environments^29–31^. The model is then used to study the motion of bacteria in the presence of a chemoattractant profile produced by a fungal network in a fluid environment. Both long-term bacterial distributions and short-term migration dynamics are modeled. We show that wrap mode increases the angular exploration of the bacteria, allowing them to move away from local maxima of the chemoattractant and explore more of the surrounding environment over time. We hypothesize that this may increase dispersion along hyphae within liquid environments. Furthermore, when bacteria are near a chemoattractant source, we find that wrap mode mainly benefits cells by increasing their angular exploration, allowing cells to move more consistently toward the source for a specific range of chemoattractant gradients. This property allows for similar aggregation speeds with lower total distances traveled compared to bacteria without wrap mode. In contrast, when bacteria are far from any chemoattractant profile, or even absent a chemoattractant gradient, our simulations suggest that wrap mode increases the rate of dispersal of *P. aeruginosa* due to larger angular changes in the direction of bacterial motion during wrap mode. Specifically, in a nonchemotactic environment, we find that an angular change of 180^∘^ during wrap mode leads to a 2.4-fold increase over dispersal without wrap mode. However, the smaller the angular change, the smaller the increase in the rate of dispersion, with angular changes of 15^∘^ even showing a small decrease in the rate of dispersion. Overall, our model highlights the critical role of bacterial reversals and wrap mode across diverse environments. Furthermore, our modeling approach is broadly applicable to other bacterial motility and chemotaxis systems, providing a framework for simulating dynamic processes in bacterial-bacterial and bacterial-fungal interactions across various species.

## Results

### Model assumptions and calibration

Based on the modeling approach introduced in ref. ^11^, we developed a subcellular element (SCE) model where each bacterium is described by five nodes connected by linear and rotational springs. The velocity of each node is then described by a cell-based SCE off-lattice submodel (see Eq. (7) in Methods section). The model was extended by adding components representing bacterial reversals and wrap mode. Descriptions of all model parameters and values are provided in Supplementary Table 1. The likelihood in the model that a bacterium enters wrap mode before reversing its direction of motion is governed by the wrapping probability *P*_*wrap*_. The bacteria will follow a run-reverse pattern when the wrapping probability is zero. For a non-zero wrapping probability, the bacteria will follow a run-wrap-reverse swimming pattern. Both run and wrap durations in the absence of a chemoattractant gradient is calibrated using the experimental data for *P. aeruginosa* presented in ref. ^14^. When a chemical gradient is present and a bacterium is moving down the gradient, the mode durations are reduced, which leads to quicker reversals. The detailed description of the calibration for run and wrap mode in the model can be found in Supplementary Note 1, and further details on how each of these features are modeled are described in the Methods section. The details of the chemotactic submodel are described in the section titled *Submodel of Chemotactic Motion*. We explore the relationship between the chemotactic sensitivity parameter *ζ*, which governs how sensitive bacteria are to changes in the concentration of the chemoattractant, and the gradient of the chemoattractant in Supplementary Note 6. For fungal simulations, the chemoattractant profile is taken from a model simulation of fungal growth. For simple chemotactic simulations, a point source of an arbitrary chemoattractant is located at the center of the domain. We use either a linear or exponential gradient, and the details of both of these calculations can be found in Supplementary Note 2.

### Wrap mode promotes bacterial exploration of complex chemoattractant profiles generated by fungi

In this section, we study how bacteria migrate in response to a chemotactic profile produced by fungi within a liquid environment. In liquid, bacteria can freely swim towards, aggregate around, and move along the fungi by responding to the various chemoattractants excreted by the fungi. Furthermore, since the area of the fungal network is relatively small compared to the area of the entire simulated domain, we make the simplifying assumption that the motion is driven entirely by chemotaxis without any mechanical interactions between the bacteria and fungi.

To test the effect of wrap mode on the motion of bacteria in the presence of a biologically relevant fungi chemoattractant profile, we used a simulated external chemoattractant field around a fungal network whose hyphal lengths are comparable to the fungus Penicillium (approximately 40 microns). For the simulated profile, the chemoattractant is excreted throughout the hyphae mass. (A figure of the model network can be found in Supplementary Note 5.) The concentration of the chemoattractant profile is in the range [1.236 × 10^−4^ mM, 2.2 × 10^−3^ mM]. The bacterial sensitivity level to chemoattractant, *ζ*, is set to *ζ*_*wrap*_ = *ζ*_*run*_ = 30 while the size of the domain is 4000 μm × 4000 μm. 10 simulations were run, leading to a total cell count across simulations of 1900 cells, where in each simulation cells were initialized in a circle surrounding the fungi, and all simulations run for a total of 1600s.

**Fig. 1:**
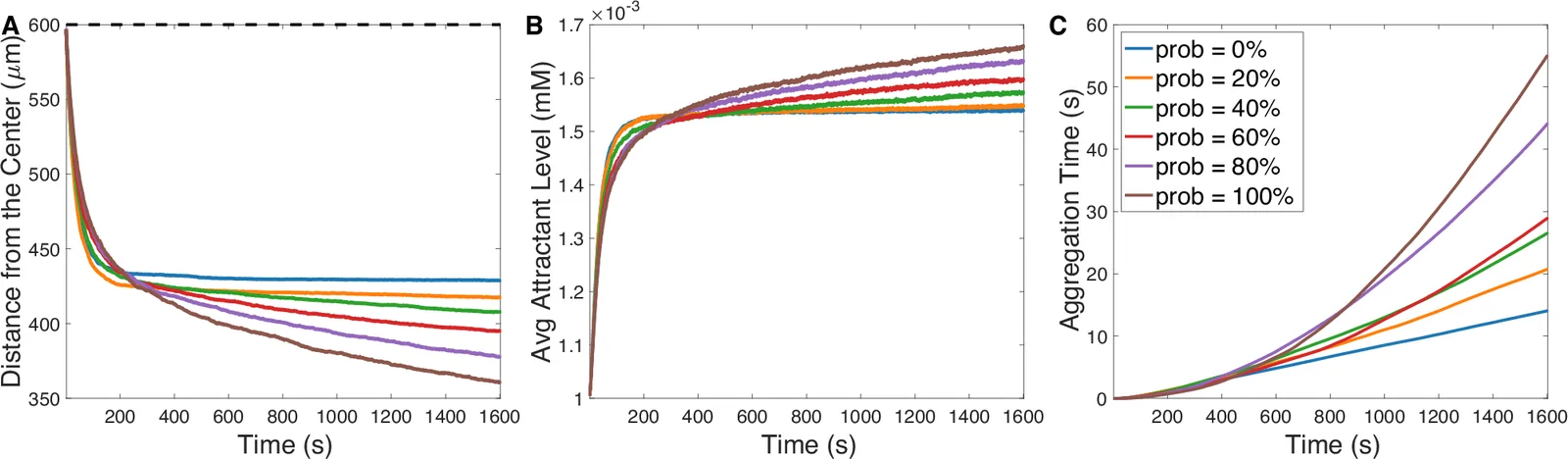
Simulations comparing bacterial migration with different probabilities of wrap mode in response to a chemoattractant profile simulated from fungal growth. **A** Shows the average distance to the center of the domain. **B** Average chemoattractant level. **C** Aggregation Time. For the aggregation time we use a radius of 200 μm which serves as a line of delineation between the tips and the center of the network, as most of the hyphae tips extend beyond 200 μm.

The center of the fungal network is positioned at the center of the domain, thus we quantify the average distance to the center of the domain between all bacteria as ${D}_{C}=\frac{1}{{N}_{b}}\mathop{\sum }\nolimits_{i}^{{N}_{b}}| | {{\boldsymbol{x}}}_{i}(t)-C| |$, where *N*_*b*_ is the number of bacteria, ***x***_*i*_ is the position of the *i*^*th*^ bacteria at time *t*, *C* is the position of the center of the domain, and ∣∣ ⋅ ∣∣ is the Euclidean norm describing the distance. Figure 1A shows that *D*_*C*_ decreases rapidly for all simulations at the beginning. However, after the bacteria approach local maximums in the chemoattractant concentrations (around 200–500 s), the change in the average distance to the center slows for all simulations. For probability of 0 and 20% of wrap mode, *D*_*C*_ flattens dramatically, leading to very little ability to continue moving toward the center as the simulation progresses past 500 *s*. However, increasing the probability past 20% increases the ability of the bacteria to continue to move toward the center. This can be seen by looking at the difference in the average distance to the center from 500s to 1600 s which is −1.86, −4.74, −14.18, −23.51, −35.36, −43.45 μm for wrap probabilities 0%, 20%, 40%, 60%, 80%, 100% respectively. This is also reflected in Fig. 1C, where we show the average time a bacteria spends within a given radius of the center/source, *τ*_*ag*_, or aggregation time. The radius was chosen to be 200 μm for this simulation. Since for lower probabilities of wrap mode the bacteria have a hard time moving after 500 s, the aggregation time is heavily influenced by whether the local maxima that the cells first reached are within 200 μm of the center. Due to the stochasticity in this process, we see that the aggregation time of 60% wrap mode actually does a bit worse than 40% in the beginning, but surpasses 40% by the end of the simulation. In general though as the probability of wrap mode increases, the ability to continue exploring throughout the simulation increases, ultimately leading to increases in the average time spent in the center circle of radius 200 μm. Finally, in Fig. 1B we show the average chemoattractant level in the local environment of the bacteria. As the probability of wrap mode increases, the bacteria’s ability to find regions of higher chemoattractants increases as well.

**Fig. 2:**
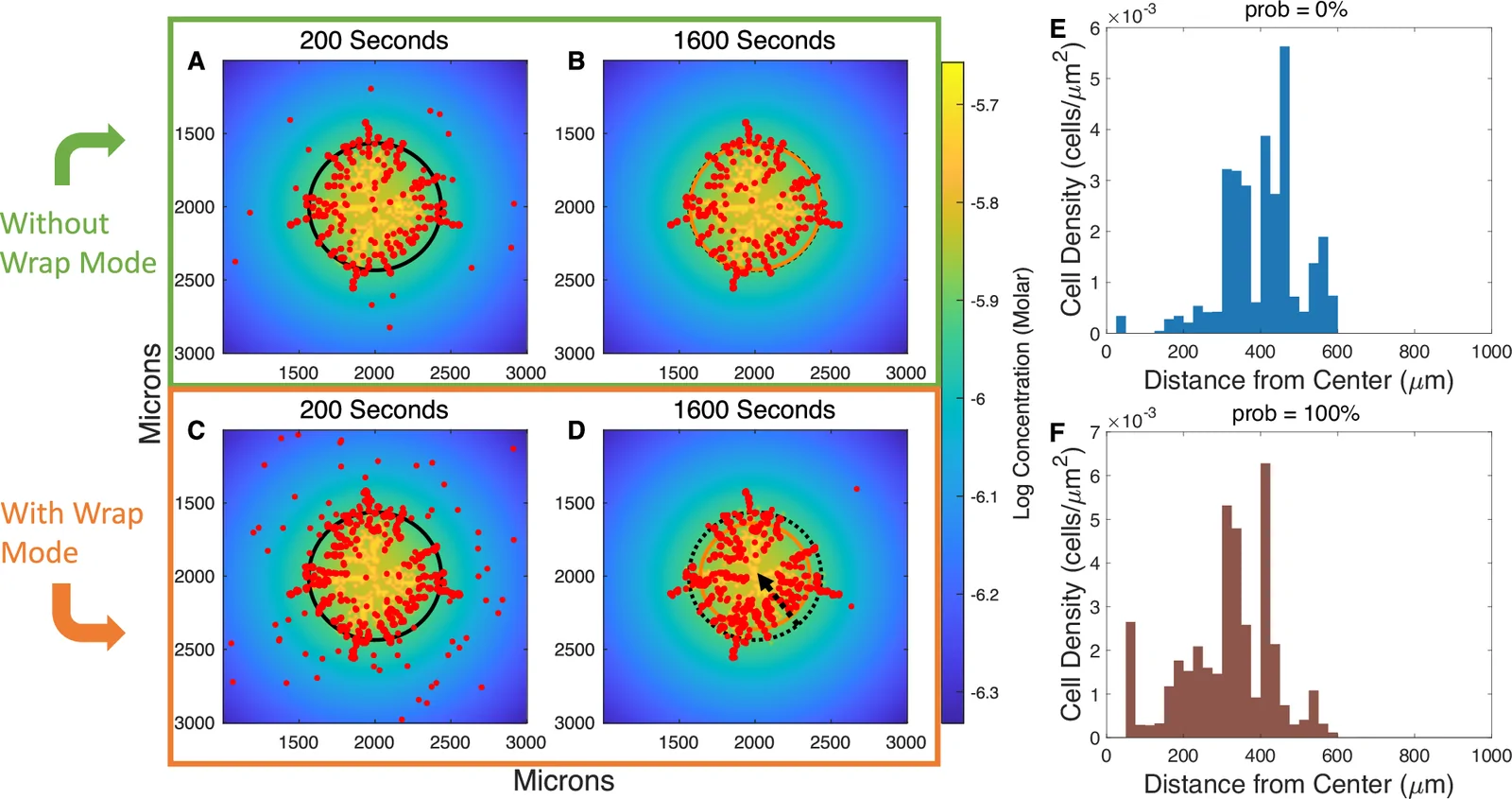
Distribution of bacteria in response to a chemoattractant profile generated from fungal growth. In **A**–**D** Bacteria are shown as red dots overlaid on chemoattractant profile which is shown in blue-yellow scale. **A**, **B** are from simulation with 0% probability of wrap and **C**, **D** are from 100% probability of wrap. **A**, **B** Show very little difference between the position of the bacteria at 200 s and 1600 s indicating that the cells are trapped at local maximums. However, with 100% probability of wrap mode (**C**, **D**) we see that the distribution has more variation between these two time steps, with cells being able to move towards the center of the network over time. For comparison, the black circle in **A**–**D** represent the average distance to the center at 200 s, while the average distance at 1600 can be seen by the orange circle in (**B**, **D**). This is further demonstrated in figures **E**, **F** where we show the distribution of the distances of the bacteria from the center of the network for 0% probability of wrap and 100% probability of wrap at the end of the simulation. We see that in **F** the distribution is shifted to the left demonstrating the increased ability to explore the chemotactic profile.

To further illustrate this property, we plot the distribution of bacteria relative to the chemoattractant profile in Fig. 2. In Fig. 2A, B we show the distributions of the bacteria at 200 s and 1600 s for the simulation with 0% probability of wrap. Here, we see that the initial maximums found by the bacteria at 200 s trap the bacteria at these locations for the entirety of the simulation. When we compare this with the distributions of the 100% wrap simulation in Fig. 2C, D, we see that cells have a greater ability to move away from their local maximum and find areas of higher chemoattractants. To quantify this we calculated the average change in distance between the cells at 200 s and at 1600 s and found that the 100% wrap mode simulation showed 745% increase in the average distance over the simulation with 0% probability of wrap.

This suggests that the increased angular exploration is an evolutionary benefit of the wrap mode. As the bacteria enter wrap mode, they can move away from a local maximum and respond to other nearby gradients. This may ultimately help bacteria explore the liquid surrounding the fungi and move toward regions of higher chemoattractants, which may be an important part of efficient migration. In order to further explore the evolutionary benefits of wrap mode, in the next two sections, we further explore the migration of bacteria when they are close to a chemoattractant source versus when they are in a low chemoattractant region.

### Wrap mode increases rate of dispersal of *Pseudomonas aeruginosa* in non-chemotactic environment

When bacteria are very far from any source of chemoattractant the concentration is low enough that the effect chemotaxis has on migration is negligible. Thus, in this section, we explore the effect of wrap mode on *P. aeruginosa* in a non-chemotactic environment. Several previous papers have explored this topic, including one study that found that wrap mode increased the dispersal rate of a related bacteria, *P. putida*^16^. In addition, for bacteria following a run-reverse pattern, it was shown that there is an optimal level of angular noise during run mode, which led to the highest effective diffusion coefficient^32^. From these studies, two competing hypotheses emerge for why wrap mode may be beneficial to bacteria in non-chemotactic environments. First, it may be that wrap mode’s primary function is to extend the time between reversals, thereby increasing dispersion rate. Alternatively, the angular change during wrap mode may coincide with an optimal level of angular noise, leading to an increased dispersion rate.

For *P. aeruginosa*, we show that large angular changes in the direction of motion of the bacteria during the wrap mode lead to an increase in the dispersion rate, while the time spent in the wrap mode is inversely related to the dispersion rate. Specifically, we show that the angular change during wrap mode leading to the highest dispersion rate is 180^∘^. Although the experimentally observed mean angle change of 90^∘^ still has a significant benefit over bacteria without wrap mode, the results are suggestive of the existence of a trade off so as to lead to the experimentally observed angle change.

In the following simulations, all durations, velocities, and reversal turn angles are chosen from the experimentally measured distributions. In 3A, B however, we decouple the duration of wrap mode from the angular change undergone during wrap mode. This is done to better understand whether the angular change or the time spent in wrap mode is more important to the diffusion of *P. aeruginosa*. Specifically, in 3A, we varied the net angular change to the cell body that occurred during wrap mode. To achieve this, we first fixed the wrapping duration by sampling the experimentally obtained distribution in ref. ^14^. Then, we calculate the direction of the force, ${F}_{\mathrm{motor}}^{0}$, at each time step needed to achieve the desired net angular change during a wrap mode of this duration (Supplementary eq. 12). Six different simulations were run to compare a run-reverse strategy vs. a run-wrap reverse strategy with wrap mode that produces angular changes of {15^∘^, 45^∘^, 90^∘^, 135^∘^, 180^∘^}. The probability of wrap mode was set to 100%. 500 cells were randomly initialized in a center square of 200 × 200 μm in a domain of size 8000 × 8000 μm and their movement was simulated for 200 seconds.

**Fig. 3:**
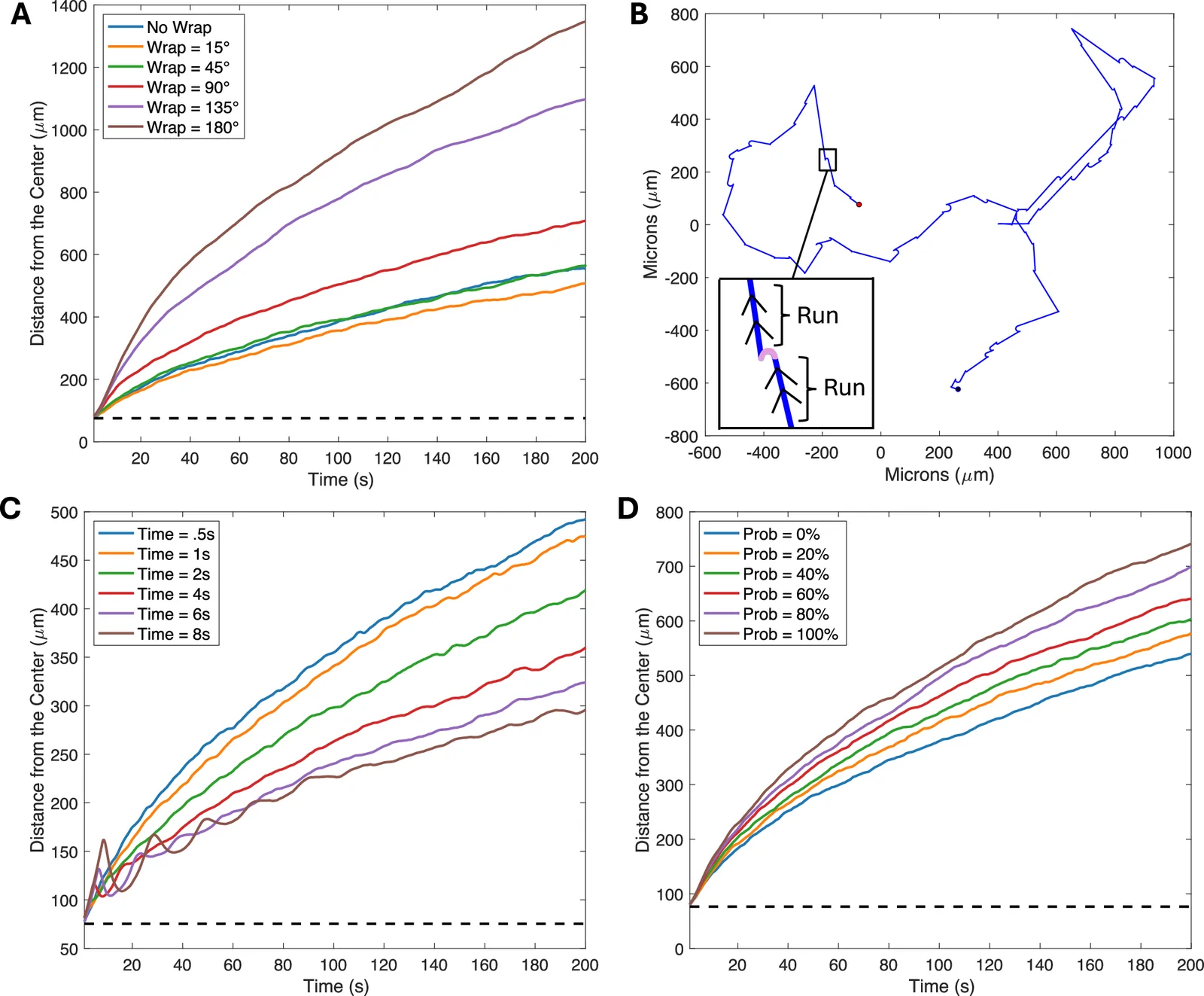
Effect of wrap angle, duration, and probability on the dispersal of bacteria in a non-chemotactic environment. Plots **A**, **C**, **D** show the average distance from the center of the domain over 200 s of simulated migration. In **A** the total angle change during wrap is varied. We see that for larger wrap angles there is a large increase in the average distance. In **C** the time spent in wrap mode is varied while the net angle change during wrap mode is fixed at 15^∘^ regardless of the time spent in wrap mode. Here *P*_wrap_ = 100%. We see that for larger wrap times we have a dramatic decrease in the average distance. In **D** probability of wrap mode is varied while the angular change during wrap is fixed at 90^∘^ and the wrap times are sampled from the experimental distributions. We see that for higher wrap probabilities we have a greater increase in the average distance. We note that a probability of 40% chance of wrap mode aligns most closely with the experimentally observed percentage of 38%. Finally **B** shows a plot of a single trajectory of a cell from figure **A** with 180^∘^ angular changes in wrap mode across the 200 simulated seconds. The red dot marks the start and the blue dot shows the ending location. The inset shows two run modes separated by a wrap mode marked in pink. Since wrap mode leads to an angular change of 180^∘^, both runs occur in roughly the same direction. For all simulations cells were initialized randomly in the central 200 × 200 μm square of a 8000 × 8000 μm domain.

Fig. 3A shows the results of the simulations. To quantify the level of dispersal, we used the average distance from the center at each time step, *D*_*C*_. For smaller angles, such as 15^∘^, there was a small decrease in *D*_*C*_ by the end of the 200 s compared to simulations without wrap mode. However, for angle changes of 90^∘^, 135^∘^, and 180^∘^, we saw a large increase in *D*_*C*_. This shows that an increase in the angular change during wrap mode leads to an increased rate of dispersal. Moreover, bacteria with a 180^∘^ angular change during wrap mode had the overall largest average distance, which represented around a 2.4-fold increase over the bacteria without wrap mode present.

To better understand why 180^∘^ produced the largest increase in the rate of dispersal, we show a typical trajectory from this simulation in Fig. 3B. We can see that a complete 180^∘^ turn right before a reversal leads the bacterium to undergo multiple runs in the same direction as shown in the inset. Thus, we conclude that this ability to have multiple runs in the same direction may be a crucial function of wrap mode and the primary driver of the increased dispersion. This finding is consistent with the hypothesis proposed in ref. ^32^ which suggested an optimal angular noise which allowed for multiple runs in the same direction might maximize dispersal.

To further explore the relationship between the time between reversals and the dispersion, we next fixed the net angle change during wrap mode and varied the total time wrap mode takes. We tested six different times of 0.5, 1, 2, 4, 6, and 8 s. The net angular change is set to 15^∘^ which allows the bacteria to continue moving in roughly the same direction as the previous run throughout the entirety of wrap mode. This is done to test the idea that wrap mode may lead to more time spent traveling in a given direction, and thus increase the dispersal of the bacteria. In Fig. 3C, we see however, that increasing the time wrap mode takes actually decreases the overall ability of the bacteria to disperse. For example, by the end of the simulation there was nearly a 200 μm difference between the average distance for the bacteria with a 0.5 s wrap mode and the bacteria with 8 s wrap mode. This is likely due to the fact that a bacterium slows down during wrap mode. Hence, the total distance traveled during the simulation decreases as the time wrap mode takes increases. This clearly demonstrates that the angle change is the driving force behind the increased dispersal and is not dependent on the time between reversal events.

Next, we computationally tested how the average distance from the center varied with different probabilities of wrap mode. We again fixed the total angular change during wrap mode as in the section above. However, since the experimentally observed mean angular change for *P. aeruginosa* during wrap mode is 90^∘^^14^, we fixed the angle change to 90^∘^ and varied the probability of wrap mode from 0 to 100%. In Fig. 3D, we plot the average distances from the center for six different probabilities. In general, we see that the larger the probability of wrap mode, the larger the average distance. Moreover, the average distance from the center was around 200μm larger by the end of the 200 s for 100% wrap mode versus no wrap mode at all. We point out that a probability of 40% aligns most closely with the experimentally observed percentage of 38%. However, other environments with different viscosities were shown to produce large variations in this probability with some viscosities even showing around 80% chance of wrap mode^14^.

### Wrap mode leads to a small increase in chemotactic efficiency at a lower total required motion

We now test the effects of wrap mode on the migration of *P. aeruginosa* when the bacteria are localized around a single chemoattractant source. Specifically, we simulated bacterial migration following a run-wrap-reverse pattern in the presence of a point source that generates a linear gradient. For all of the following simulations, we set the chemotactic sensitivity to be the same in both modes, *ζ*_wrap_ = *ζ*_run_ = 1, and compared the chemotactic efficiency of 0% probability of wrap mode vs. 38%, which is the experimentally observed rate of wrap mode found in ref. ^14^. We note that the chemotactic sensitivity controls how responsive the bacteria are to the chemoattractant gradient, with larger sensitivity values resulting in quicker reversals when the bacteria move down the gradient (see Eq. (9)). Chemotactic efficiency is quantified by the following three metrics: the average distance to the source *D*_*S*_, directional efficiency, defined as the percentage of the total distance traveled that is directed toward the source, and aggregation time *τ*_*ag*_, with a given radius of 50 μm. 1000 bacteria were initialized in a circle centered around the source of the chemoattractant on a domain of 4000 × 4000 μm. The slope of the chemoattractant profile was set at 2. We tested three scenarios that differed in the initial orientation of the cell body relative to the chemoattractant gradient. This is done to help identify whether the benefits of wrap mode are primarily due to increased ability of the bacteria to reorient their motion toward the source.

**Fig. 4:**
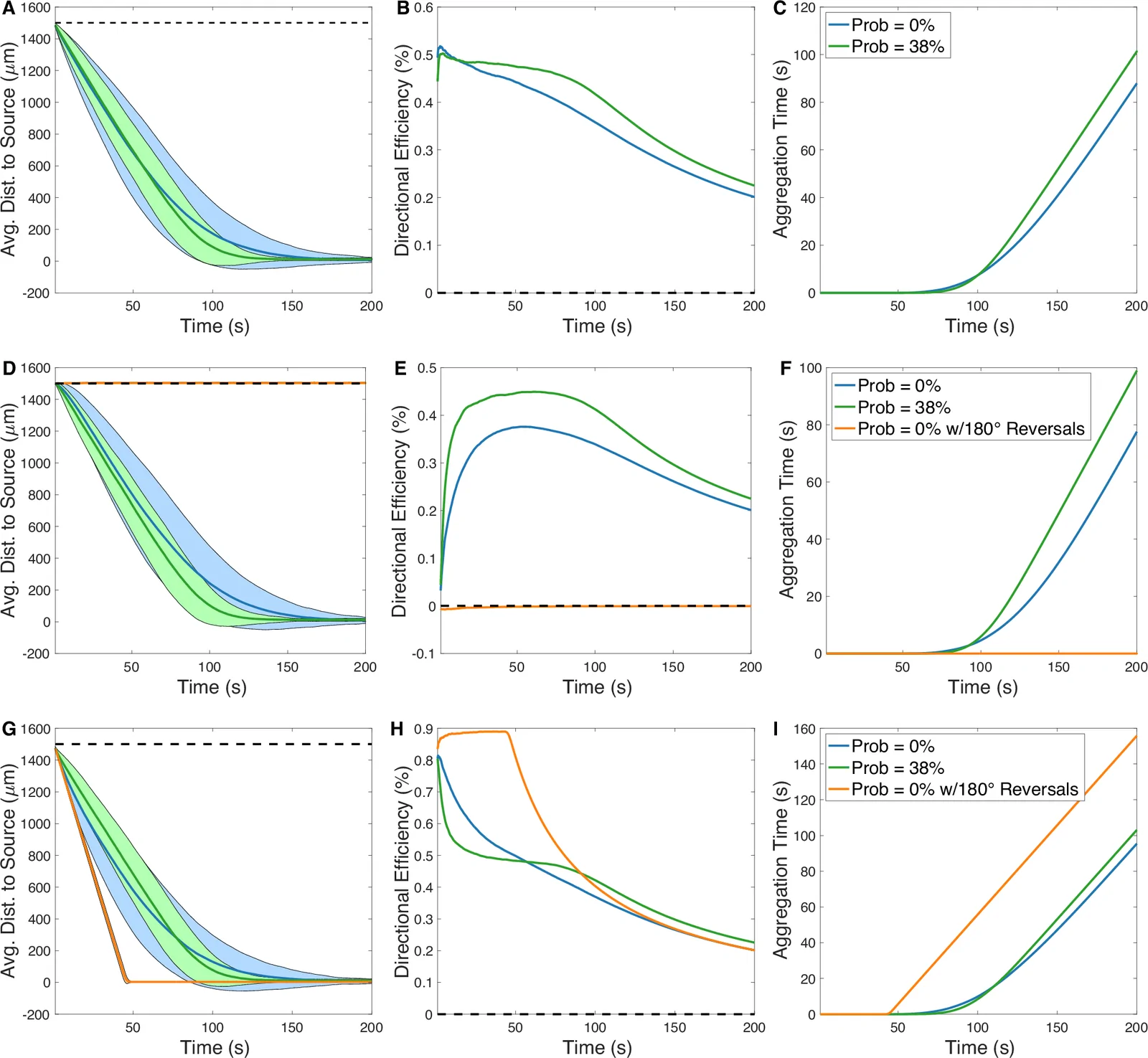
Simulations comparing bacterial migration with 0% probability of wrap and 38% which is the experimental observed probability of wrap mode^14^. Cells are initially oriented randomly in (**A**–**C**), perpendicularly (**D**–**F**), and parallel (**G**–**I**) with respect to the gradient. We see that when cells start with their initial orientation perpendicular to the gradient, the difference between a run-reverse strategy and run-wrap-reverse strategy becomes more pronounced. In contrast when the cells are already pointed toward the source there is very little difference between the migration strategies suggesting the increased ability to reorient the cell during wrap mode is one of wrap modes main benefit.

We first initialized the bacteria with random orientations of their cell bodies relative to the gradient. In Fig. 4A, we see that for the first 55 s of the simulation, *D*_*S*_ is roughly the same for both 0% and 38% probability of wrap mode. However, after 55 s, we start to see that 38% performs slightly better as all the bacteria find and localize around the source. This leads to a maximum difference of 85.43 μm between the two average distances which occurs at 96.9 s. We can also see that the standard deviation, as depicted by the green and blue shading, is greater for 0% wrap than for 38% probability of wrap. Furthermore, by 140 s we see that almost all cells for the 38% probability of wrap simulation have found the center of the source while the standard deviation for 0% is still 85.53 μm. In addition, Figure 4C shows that *τ*_*ag*_ is greater for 38% probability of wrap when compared to 0% probability of wrap. By the end of the simulation, the bacteria with a 38% probability of wrap mode spent on average 13.41 s more inside 50 μm of the center than the bacteria without wrap mode. Both observations suggest that, while there is a small difference in the averages, wrap mode increases angular exploration, helping the bacteria swim more consistently up the gradient.

In Fig. 4B, we plot the percentage of the total distance traveled by the cell that is directed toward the source. We call this metric directional efficiency. We note that given two hypothetical strategies with the same chemotactic efficiency, the strategy with less overall motion will have a larger directional efficiency since bacteria are able to achieve the same aggregation with less total motion. Thus, this metric helps quantify any metabolic benefit afforded by the chemotactic strategy. We see that with a 38% probability of wrap, more of the total motion of the bacteria is directed toward the source. The maximum difference occurs around 91 *s* with the values being 43.61% and 37.46% for wrap vs. no wrap, respectively. This indicates that with wrap mode, bacteria are able to move up the gradient, with nearly 6.2% more of their overall motion, contributing to their movement towards the source.

We point out that this increase in chemotactic efficiency, as outlined above, occurs only within a limited range of chemoattractant gradients. In Supplementary Note 6, we show the same plots for chemoattractant profiles with slopes of $\frac{1}{2}$, 1, 2, 4. The results suggest that the outcomes shown above are partially dependent on the strength of the gradient. For example, we see that for slopes less than $\frac{1}{2}$ the run-wrap-reverse pattern performs worse than the run-reverse strategy. This is the case because wrap mode increases the dispersal of the bacteria in low-gradient environments. If the gradient is not large enough to meaningfully impact the run and wrap durations, then wrap mode only serves to increase the dispersal of the bacteria, which does not correspond to an increased chemotactic efficiency. In contrast, since bacteria in model simulations update their reversal frequencies every .1 seconds, using Eq. (9), we see that as *v* ⋅ ∇ *s* < 0 decreases − *ζv* ⋅ ∇ *sΘ*( − *v* ⋅ ∇ *s*) → *∞*. Thus, the reversal frequency becomes increasingly large, inducing a reversal almost immediately when moving down the gradient. In this case, any increased angular exploration from wrap mode can ultimately be achieved by repeated reversal events given a strong enough gradient.

Next, we examine how the initial orientation of the bacteria affects the above results. To do this, we ran the same set-up twice and compared the results when the bacteria are all initially oriented perpendicularly to the gradient versus parallel. In Fig. 4D–F, we show the results for bacteria that are initially oriented perpendicularly. We see that when the bacteria are all facing away from the gradient, there is an increased separation between 0 and 38% probability of wrap for each of the three statistics discussed above. In particular, we now see that 38% probability of wrap performs slightly better than 0% probability of wrap with respect to *D*_*S*_ and the difference between the two increases throughout the first 100 s. This difference shows the importance of wrap mode in reorientation. Since all the motion in Fig. 4D is directed away from the source at the start, the bacteria without wrap mode have a harder time reorienting and moving up the gradient. As a base comparison, we also plot the migration of bacteria that undergo a run-reverse pattern, but where the angle change in their reversals are strictly 180^∘^. We see that since the bacteria are oriented perpendicularly, they are not able to move any closer to the source and thus remain stuck throughout the whole simulation. In contrast, when we initially orient the bacteria towards the source, as in Fig. 4G–I, we see that the 0% probability of wrap performs slightly better up until 75–80 s. After this point, both the percentage of total distance traveled in the radial direction, as well as the average distance from the source for 38% probability of wrap finally overtakes 0% probability of wrap. Again, we show the hypothetical third motility strategy, in which the bacteria undergo 180^∘^ reversals. Here, we see that this strategy vastly outperforms both of the other two methods. This illustrates that when reorientation is not required, the best strategy limits the angular variation. However, in biologically realistic settings, being able to reorient becomes crucial to efficient aggregation.

### The experimentally observed frequency of wrap mode may represent an optimal trade-off between chemotactic efficiency and total motion required for migration

**Fig. 5:**
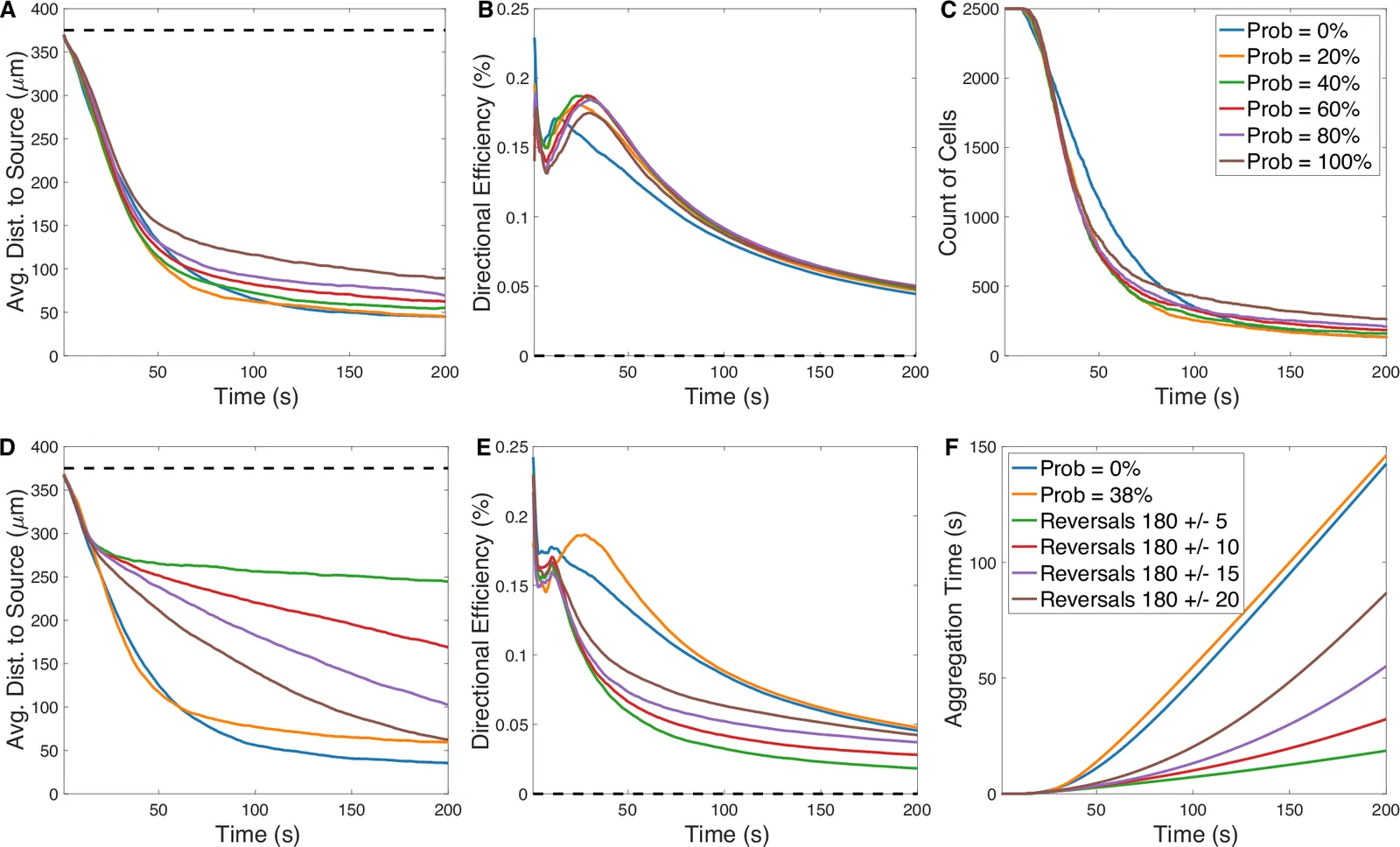
Effect of wrap probability and reversal angles on aggregation of bacteria in response to an exponential gradient. **A**–**C** Compare bacterial migration with different percentages of wrap mode, while **D**–**F** compare a run-wrap-reverse strategy to a run-reverse strategy with various restrictions to the angle changes that occur during reversals. **A**, **D**, average distance to the source; **B**, **E**, percentage of the total distance traveled directed towards the source; **C**, number of cells with a distance from the source greater than 50 μm; and **F**, aggregation time.

While diffusion of a single bolus of a chemoattractant from a source often results in a linear gradient over long periods of time, continuous production of a chemoattractant from a fungi would be expected to generate a sustained exponential gradient. Here, we evaluate the bacterial migration in response to a more realistic exponential gradient that would be expected from an actively metabolizing species and further examine the two proposed benefits of wrap mode. We set a point source in the center of a 2000 × 2000 μm domain and initialized bacteria in a circle of radius 375 μm with random orientations with respect to the gradient. The source is generated from a reaction-diffusion equation with the parameters listed in Supplementary Table 1. The chemotactic sensitivity is set to *ζ*_wrap_ = *ζ*_run_ = 20 and 2500 cells are simulated for 200 s. In Fig. 5A–C this is done for each percent probability of wrap mode {0%, 20%, 40%, 60%, 80%, 100%}. In Fig. 5A, we see that during the first 45 s, all percentages of wrap mode perform very similarly as measured by *D*_*S*_. However, after 45 s we see that *D*_*S*_ begins to level out with 0% and 20% being the smallest and increasing as the probability increases. In Fig. 5C, we plot the number of cells that are further than 50 μm from the center. From this plot, we see that the number of cells is significantly fewer for wrap mode simulations before the 80 s mark, demonstrating an increased ability for cells to find the source. However, by the end of the simulated time, we see that 0% probability of wrap ultimately overtakes the wrap mode simulations in both Fig. 5A, C. Part of the reason for this is due to the nature of the exponential gradient yielding a low chemoattractant environment where the cells are initialized. As discussed above, wrap mode actually increases the dispersion in low-gradient environments. For this reason, some of the cells move away from the source at the beginning and get trapped in areas of low chemoattractant. This can be seen in the number of cells that are still farther than 50 μm from the center. At 200 s there are 137, 135, 161, 185, 210, and 264 cells for wrap probabilities 0%, 20%, 40%, 60%, 80%, 100% respectively. This shows that as wrap mode increases, the likelihood of being stuck in low-gradient environments increases.

In Fig. 5B, we see that for the beginning of the simulation, 0% probability of wrap mode outperforms the wrap mode simulations very briefly. This is again likely due to the initial period of increased exploration with wrap mode in the low-gradient regions. However, after the initial 20 seconds, all the wrap mode simulations overtake the no-wrap simulation. Furthermore, we see that the simulations with a 40−80% probability of wrap mode perform very similarly with respect to the percentage of the total distance traveled that is directed toward the source. This is likely due to two opposing outcomes of wrap mode. First, wrap mode slows down the bacteria, so an increased probability leads to less total distance traveled. However, wrap mode also increases the angular exploration, which may be counterproductive at a high enough percentage. This suggests that the experimentally observed percentage of 38% may provide an optimal trade-off between a metabolic benefit and overall chemotactic efficiency. We again note that these benefits only occur within a range of gradient/sensitivities. For low and high gradients/sensitivities, wrap mode is likely not to provide as much added benefit.

### Limiting the distribution of angle changes in reversal events drastically reduces the overall chemotactic efficiency

Finally, we examine how much angular variation is needed for efficient migration. To test this, we looked at what happens to the chemotactic efficiency when we limit the angular changes from reversal events. Here, we compare both 0% and 38% probability of wrap to the simulated motility of bacteria where angle changes during reversals are limited to 180 ± 5^∘^, 10^∘^, 15^∘^, 20^∘^. This is done by repeated sampling of the angle of rotation in the force on the frontier node at each time step, to ensure that the chosen direction produces a reversal angle in the designated range. In Fig. 5D–F, we can see a clear trend. Here, 38% probability of wrap mode leads to the best aggregation at the beginning of the simulation and then *D*_*S*_ flattens out above that of the run-reverse strategy showing that with wrap mode the cells still maintain some increased exploration around the point source. When the angular variation during reversals is reduced, we see that the chemotactic efficiency becomes worse the less angular variation exists. This further confirms that most of the benefits of wrap mode likely comes from an increase in angular exploration. However, since wrap mode only marginally improves the average distance to the source and aggregation time, this also suggests that the distribution of angle changes in reversal events might be important to the overall migrational efficiency, as well.

## Discussion

In this paper, a novel computational model of *P. aeruginosa* is used to explore the impact of reversals and wrap mode on bacterial migration with and without chemotaxis in different environments. First, we studied the effect of wrap mode on bacterial migration in response to a chemoattractant profile produced by a fungal network in liquid. We found that increasing the probability of wrapping mode led to a greater ability of bacteria to explore their local environment. Compared to a run-reverse strategy alone, a run-wrap-reverse strategy increased the likelihood that the bacteria could move away from local chemoattractant maximums and find nearby regions with a higher concentration of chemoattractant. This is in part due to the sensitivity of the chemotactic response of each bacterium. For very weak chemotactic responses and for very strong chemotactic responses where reversals are induced almost immediately when swimming down the gradient, it is likely that run-reverse and run-wrap-reverse have similar outcomes. However, when the chemotactic response is strong enough to keep bacteria close to the source, but allow for some movement, the introduction of wrap mode introduces additional run time and angular exploration, which allows cells to overcome the chemotactic pull of a local maximum and explore other parts of the profile. On the basis of this, we hypothesize that the wrap mode may help bacteria to explore multimodal chemoattractant profiles like those produced by hyphal networks in liquid.

To better understand this phenomenon, we then further studied the migration of *P. aeruginosa* both in the absence of chemotaxis and when the bacteria are near one of the source points along a fungi. In the absence of chemotaxis, it was shown that wrap mode enhances the ability of the bacteria to disperse across a fluid environment. However, this was dependent on the angle of rotation of the cell body that occurred during wrap mode. When wrap mode induced smaller angular changes to the bacteria’s direction of motion, a minor decrease in the dispersal was observed in the model simulations. In contrast, larger angular changes during wrap mode (90^∘^, 135^∘^, and 180^∘^) resulted in a significant increase in the dispersal of the bacteria. This finding suggests that wrap mode is instrumental in increasing the angular exploration of the bacteria and thereby their dispersion, which has been shown to be a crucial factor in both bacteria’s ability to locate sources of nutrients^33^ as well as the spatial composition and spreading of biofilms^34^.

With chemotaxis present, we examined the effects of wrap mode on bacterial migration in response to both linear and exponential chemotactic source. We found that wrap mode had a minimal effect on the migration of the bacteria with respect to the average distance to the source. Instead, our results show the primary function of wrap mode is to increase the angular exploration. Due to this, we find that wrap mode has varying effects depending on the chemotactic strength. With low chemoattractant gradients or low chemotactic sensitivities, a run-wrap-reverse strategy performs slightly worse than a run-reverse strategy. Since wrap mode increases the angular exploration of the bacteria, if chemotaxis is relatively weak, this exploration is not strongly biased toward the source of the chemoattractant and only serves to slow down the bacterium without contributing to the chemotactic efficiency. In contrast, when the chemotactic force becomes strong enough, exploration away from the source is reduced, causing wrap mode to increase the chemotactic efficiency. In this range, we find that the wrap mode reduces the total distance traveled that is needed to achieve similar aggregation speeds and allows bacteria to move up the gradient more consistently. Thus, we hypothesize wrap mode may reduce the metabolic expenditure of the bacteria required for migration and may make the bacteria more responsive to changes in the chemoattractant profile.

We note that the conclusion of this study differs some from the results in ref. ^14^ where simulations suggest that wrap mode increases the average displacement of *P. aeruginosa* by around 20%. One possibility for this variance is the difference in the chemotactic submodel used between the two models. In ref. ^14^, a Monod-Wyman-Changeux model of chemotactic response is used, which includes adaptation, or the tendency of the chemotactic response to gradually reset and adapt to the levels of chemoattractants in the local environment over time. This allows the bacteria to be sensitive to a large range of gradients. Although this is a well documented phenomenon, we note that regardless of the chemotactic sensitivity explored in this paper or whether the chemoattractant profile was linear or exponential, wrap mode did not show the same increase in average displacement found in ref. ^14^. Furthermore the main finding that wrap mode does help increase the exploration of bacteria in liquid environments in the presence of multi-model profiles, like those seen from fungi in liquid environments, suggests that the benefits of increased angular exploration represent an additional part of the possible evolutionary basis for wrap mode.

However, it should be noted that the advantages related to wrap mode under chemotactic conditions in the model appear to be subtle and may not actually be experimentally significant. Although the model parameters were calibrated based on experimental phenomena, different parameter values may produce qualitatively different results. In fact, setting the model’s internal force field (spring constant) to different values so as to adjust the flexibility of the bacterium, results in qualitatively different dynamics during reversal events and wrap mode when angular changes occur. However, since these angular distributions were calibrated to the experimental data in ref. ^14^, any change in the spring coefficient would only lead to the recalibration of other forces so as to recover the same distributions and ultimately the same trajectories. Please refer to the SI, where we showed it was possible to recover the same trajectories given a different bending coefficient.

In addition, one other point of consideration is that of the introduction of noise within run-mode itself. Although some previous studies have found that the migration of certain bacteria can be modeled as a diffusive process, it is important to distinguish what has been referred to as “active" diffusion and rotational diffusion as a result of thermal fluctuations. For instance in ref. ^35^ it was found that tumbles in *E. coli* can be modeled as rotational diffusion. However, rather than the tumbling being caused by thermal fluctuations, the term “active" alludes to this being a property of the flagellar activity. For this reason, in chemotactic settings, some authors have chosen to neglect rotational diffusion as it is independent of the ligand concentration and is subdominant in the chemotactic process^36^. While *P. aeruginosa* is a singularly flagellated bacteria which does not tumble, and largely swims in a straight line during runs, in Supplementary Note 7, we show how the results presented above in Fig. 4 are impacted depending on the level of noise introduced during each run mode. In general we found that for the levels tested, the greater the amount of noise, the less differences between run-wrap-reverse and run-reverse strategies in linear gradients. This further supports the hypothesis that wrap mode serves a key role of angular exploration as the noise allows for more angular exploration without wrap mode. However, it does highlight the benefit of having the extra time/phase where this exploration occurs through wrap mode. Since the exploration done in wrap mode extends the motion before a reversal, it is likely that this is instrumental to their ability to escape local maximums in a multi-modal profile.

Given these considerations and those above discussing varied responses across chemotactic sensitivities, whether wrap-mode for bacteria in liquid environments actually confers a selective advantage experimentally is not clear. Our results do however, suggest that wrap mode may be most important in structured environments where increased angular exploration is necessary in navigating the environment. Furthermore, the developed modeling environment can be used for studying bacterial-fungi interactions for a variety of different species, and the model is currently being extended to the case of growing fungi network. Moreover, the model provides a versatile platform for studying bacterial-fungal interactions across a variety of species, enabling researchers to discover species-specific behaviors and mechanisms of interaction^2,37^. By incorporating simulations with various environmental conditions, such as variations in pH, temperature, and nutrient availability, this model can provide valuable insights into how these interactions adapt to or are influenced by ecological pressures^2,37^. Lastly, our model could provide the framework for dynamic modeling of fungal network growth in the presence of bacteria, allowing detailed study of how bacterial interactions influence fungal network density and resource distribution over time^38^.

## Methods

In this section, we describe an agent-based SCE sub model representing the mechanical properties of each individual bacterium and the mechanical forces that act on the bacterium. Previously, the SCE approach was used for simulating swarming of the bacterium *Myxococcus xanthus*^11^ and *P. aeruginosa*^26^. The SCE model is coupled in this paper to a novel chemotactic sub-model, which modulates the reversal frequency of each bacterium and direction of its motion. The coupled model simulates the ability of *P. aeruginosa* to respond to various chemical profiles.

### Model of *P. aeruginosa*

In the subcellular element (SCE) modeling approach used here, each cell is represented by a sequence of nodes connected by linear and rotational springs. Linear springs are used to model the bonding energy between consecutive nodes, which allows for some strain in the length of the bacterium, while also maintaining the average length over time. Meanwhile, rotational springs are used between three consecutive nodes, which allows the cell body to turn and change directions over time.

We chose to model bacteria as elongated rods which can bend through the use of the Subcellular Element Model (SCE) primarily because elongated shape is biologically more realistic than representing bacteria as brownian particles or rigid rods. The average length of *P. aeruginosa* is between 1 and 5 micrometers^39^, which matches the scale of each modeled cell in our simulations. Furthermore, studies have shown that bacterial cell walls deform under transient mechanical forces. In particular it was demonstrated in ref. ^40^ that bacteria bend momentarily under certain forces, and then fully recover their original shape once the force is removed. Furthermore, in situations where sustained forces are applied during active cell wall growth, the bacteria can even undergo plastic deformation^40^.

Although the ability to bend might not be immediately necessary to reproduce experimental phenomena of freely swimming bacteria in fluid, a minimal model is not necessarily a better model, especially if it lacks realism. Moreover, the elongated representation is an important component in the modeling of bacteria, allowing for other questions to be addressed in the future where cell geometry is important. These include cell-cell interactions, bacterial wrap mode motion in viscous fluid and restricted spaces such as bacterial navigation around fungi in soil^41^, or the mechanical deformation of bacteria and bacterial envelopes upon surface adhesion^42^. In addition, we also model bacteria as elongated rods to add in the future biologically relevant distribution of type Pili IV along the bacterial body^26^, since they play an important role in bacteria-fungi interactions^43–45^. Finally mechanical properties of bacteria including stiffness under different conditions, can be calibrated in our model using recent AFM measurements^46^.

For the reasons mentioned above, we model each cell to be of length 2 μm. To maintain reasonable computational costs while still accurately modeling the physical forces on each cell, we represent each bacterium by a sequence of five nodes. Thus, the distance between each node is ${l}_{eq}=\frac{2}{{N}_{nodes}-1}=0.5\,\mu m$. The bonding force ${{\boldsymbol{F}}}_{\mathrm{bond}}^{i}$ on node *i* at location ***x***_*i*_ is calculated by the expression.

1 $${{\boldsymbol{F}}}_{\mathrm{bond}}^{i}=\sum _{j\in BN(i)}{k}_{b}(\parallel {{\boldsymbol{x}}}_{i}-{{\boldsymbol{x}}}_{j}\parallel -{l}_{eq}){\hat{{\boldsymbol{r}}}}_{i}$$

The set of bonded neighbors of node *i* is denoted by *BN*(*i*), *k*_*b*_ is the linear spring constant, and ${\hat{{\boldsymbol{r}}}}_{i}$ is the unit vector pointing from node *i* to node *j*. To determine the bending force^47,48^ on each joint comprised of 3 consecutive nodes, we first write the bending energy *U*_bend_ between the nodes *i*, *j*, and *k*

2 $${U}_{bend}={k}_{bend}[1-\cos ({\theta }_{ijk}-{\theta }_{eq})]$$

where *k*_bend_ is the rotational spring constant, *θ*_*ijk*_ is the angle between nodes *i*, *j*, and *k*, and *θ*_*eq*_ = *π* rad is the equilibrium angle. The bending forces of a joint (${{\boldsymbol{F}}}_{bend}^{i},{{\boldsymbol{F}}}_{bend}^{j}$, and ${{\boldsymbol{F}}}_{\mathrm{bend}}^{k}$) can then be calculated by taking the derivative of the bending energy as follows^49^.

3 $${{\boldsymbol{F}}}_{{bend}}^{i}=-{k}_{{bend}}\left(\frac{{{\boldsymbol{r}}}_{kj}}{| {{\boldsymbol{r}}}_{ij}| | {{\boldsymbol{r}}}_{kj}| }-\cos\,{\theta }_{ijk}\frac{{{\boldsymbol{r}}}_{ij}}{| {{\boldsymbol{r}}}_{ij}{| }^{2}}\right)$$

4 $${{\boldsymbol{F}}}_{{bend}}^{k}=-{k}_{{bend}}\left(\frac{{{\boldsymbol{r}}}_{ij}}{| {{\boldsymbol{r}}}_{ij}| | {{\boldsymbol{r}}}_{kj}| }-\cos\,{\theta }_{ijk}\frac{{{\boldsymbol{r}}}_{kj}}{| {{\boldsymbol{r}}}_{kj}{| }^{2}}\right)$$

5 $${{\boldsymbol{F}}}_{\mathrm{bend}}^{j}=-({{\boldsymbol{F}}}_{\mathrm{bend}}^{i}+{{\boldsymbol{F}}}_{\mathrm{bend}}^{k})$$

where ***r***_*ij*_ = ***x***_*i*_ − ***x***_*j*_. Furthermore, in order to simulate the propulsive force applied by the flagellum on the cell body, we introduce a force ${{\boldsymbol{F}}}_{motor}^{i}$ on node *i*. The magnitude of the force, *f*_motor_ is calibrated to the velocity in both run and wrap mode. The details are found in Supplementary Note 1. To smooth the motion of the bacterium, the propulsive force is distributed uniformly among all five nodes. Hence the force, ${F}_{motor}^{i}$, applied to node *i* is calculated by the equation

6 $${{\boldsymbol{F}}}^{{i}_{motor}}={f}_{motor}{\hat{{\boldsymbol{r}}}}_{i-1,i},\quad 1\le i\le 4$$

where ${\hat{{\boldsymbol{r}}}}_{i-1,i}$ is the unit vector pointing in the direction ***r***_*i*−1,*i*_. In the absence of a reversal event or additional forces, the force on the head node is ${{\boldsymbol{F}}}_{\mathrm{motor}}^{0}={f}_{\mathrm{motor}}{\hat{{\boldsymbol{r}}}}_{0,1}$. The motion of each node is represented by a cell-based SCE off-lattice sub model. We make the assumption that the viscosity of the fluid is large enough such that the inertial forces can be discounted. In addition, we make the assumption that the liquid environment is completely homogeneous and without any flow; thus the propulsive force from the flagella will dominate the bacterium’s motion. Furthermore, any stochastic elements from thermal fluctuations can be accounted for through the stochasticity in the reversal frequencies, as well as angle changes in reversal events and during wrap mode. Finally, each bacterium is represented by five nodes which only accounts for a coarse-grained representation. Therefore, the equation of motion is written as follows

7 $$\frac{d{{\boldsymbol{x}}}_{i}(t)}{dt}=-\frac{1}{\gamma }{\nabla }_{x}U({{\boldsymbol{x}}}_{i}(t))$$

where *γ* is the friction coefficient and − ∇_***x***_*U*(***x***_*i*_(*t*)) is the net force acting on node *i* which can be written as

8 $$-{\nabla }_{x}U({{\boldsymbol{x}}}_{i}(t))={\boldsymbol{F}}({{\boldsymbol{x}}}_{i}(t))={{\boldsymbol{F}}}_{bond}^{i}+{{\boldsymbol{F}}}_{bend}^{i}+{{\boldsymbol{F}}}_{motor}^{i}$$

In Eq. (7), the left-hand side represents the velocity of node *i* at position ***x***_*i*_, where as the right-hand side accounts for the bonding, bending, and motor force applied to this node. Equation (7) is solved using a forward Euler method. Furthermore, we impose periodic boundary conditions on the 2D simulated domain. This is implemented by identifying opposite edges of the domain together, so that if a bacterium moves off of one side it reappears on the other. In this way, the total number of bacteria is maintained throughout the simulation.

### Reversal events and mode durations

Between pull and push mode, *P. aeruginosa* performs a reversal in which the orientation of the cell body undergoes a rapid change, typically between 90^∘^ and 180^∘^. This process is modeled by first reversing the order of the nodes, such that the frontier node becomes the rear node, and then altering the direction of the force on the frontier node for 0.2 seconds. The direction is selected randomly from a distribution such that the net angle changes for all reversal events match the experimentally observed distribution in ref. ^14^. Furthermore, the durations for both run and wrap mode can be written as ${T}_{run}=\frac{1}{{\lambda }_{run}}$ and ${T}_{\mathrm{wrap}}=\frac{1}{{\lambda }_{\mathrm{wrap}}}$ where *λ*_run_ and *λ*_wrap_ are the reversal frequencies in run and wrap mode, respectively. The initial durations *T*_run_ and *T*_wrap_ are selected from the calibrated distributions at the beginning of each mode. Each bacterium is then given an internal clock which increases with every time step. Once the mode duration is met, the bacteria either reverses or enters wrap mode. The details of the reversal angle and mode duration calibrations can be found in Supplementary Note 1.

### Wrap mode

**Fig. 6:**
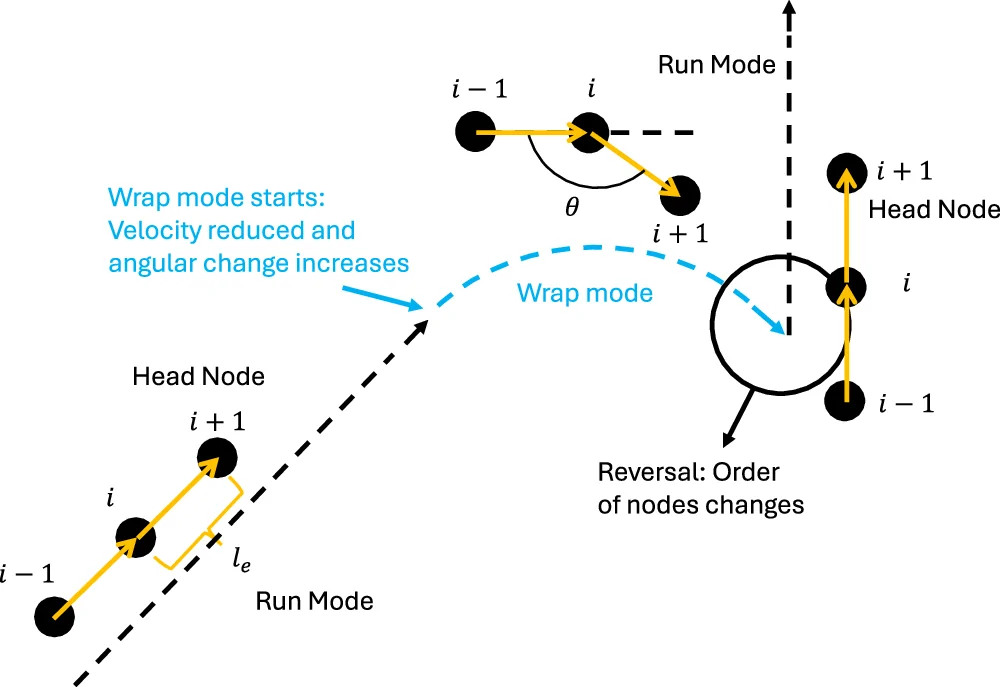
A simplified diagram of the SCE model which represents each cell as three nodes connected by springs. We show a typical trajectory of a cell undergoing a run-wrap-reverse pattern.

The defining characteristics of wrap mode are a reduction in cell velocity, when compared to run mode, and an increase in the angular velocity. In accordance with^14^, we define a probability for each bacterium to enter wrap mode before it reverses. Once in wrap mode, the propulsive force *f*_motor_ is reduced to match the experimentally observed velocity in wrap mode. Wrap mode also delays the reversal events described above. In wrap mode, the internal clock will be reset, and once the selected duration for wrap mode has been met, the bacterium will undergo a reversal event. To obtain an angular rotation during wrap mode, the direction of ${F}_{\mathrm{motor}}^{0}$ is changed from ${\hat{{\boldsymbol{r}}}}_{0,1}$ to $\hat{{\boldsymbol{w}}}$ where $\hat{{\boldsymbol{w}}}$ is chosen from a distribution of possible directions. This distribution, as well as the durations of the wrap mode, are calibrated to match the statistics reported in ref. ^14^ and the details can be found in Supplementary Note 1. A simple diagram of SCE components of the model with only three nodes making up each bacteria can be seen in Fig. 6.

### Submodel of chemotactic motion

Each bacterium is able to modulate its motility to navigate to regions of higher metabolites. In general, a bacterium accomplishes this by adjusting its reversal frequency, which results in the bacterium spending more time moving up the gradient of a chemoattractant, rather than down the gradient. We model the reversal frequency by the following equation, which follows previous work on bacterial migration^50^.

9 $$\lambda ={\lambda }_{0}{e}^{-\zeta (v\cdot \nabla s)\Theta (-v\cdot \nabla s)}$$

10 $$\Theta (u)=\left\{\begin{array}{ll}0\quad &\,\mathrm{for}\,u < 0\\ 1\quad &\,\mathrm{for}\,u\ge 0 \end{array}\right.$$

where *λ*_0_ is the rate of reversal in the absence of chemoattractant, *v* is the velocity of the bacteria. The concentration of the chemoattractant is represented by *s*, and *ζ* is a parameter that governs the sensitivity of the chemotactic response. Since *Θ* is the Heaviside function, the bacteria are considered pessimistic in their chemotactic response. This means that when a bacterium is moving up a gradient we have − *v* ⋅ ∇ *s* < 0 and *Θ* = 0. Therefore the reversal frequency remains unchanged allowing the bacterium to continue moving up the gradient. On the other hand, when a bacterium is moving down the gradient we have that − *v* ⋅ ∇ *s* > 0 and *Θ* = 1. This results in an increase to *λ* which causes the bacteria to reverse quicker and move in another direction that may be more beneficial. We note that the durations of run and wrap mode are modulated by Eq. (9) since the duration is the inverse of the reversal frequency as discussed in the Section titled *Reversal Events and Mode Durations*.

### Implementation of chemoattractant profile

The profile of the chemoattractant is stored on a discretized domain with a grid size of 1 μm. The size of this grid is chosen to be smaller than the distance traveled between consecutive updates in the chemotactic submodel. Since the gradient is checked every 0.1 s, and the bacterial velocity is $14.45\,\frac{\mu m}{s}$ in wrap mode, the grid size must be smaller than 1.45 μm. Furthermore, we assume that the bacteria sense the gradient by comparing their current environment to the environment at the previous time point. Thus, we calculate the term *v* ⋅ ∇ *s* used for a bacterium at time *t* and position ***x*** as

11 $$v\cdot \nabla s=\frac{s({\boldsymbol{X}}(t+\bigtriangleup {t}_{\zeta }))-s({\boldsymbol{X}}(t))}{\bigtriangleup {t}_{\zeta }}$$

where ***X*** is the nearest grid point to the off-lattice coordinate ***x***, and △*t*_*ζ*_ is the time between updates to the chemotactic model. We assume that the time scale of diffusion of the chemoattractant is larger than that of the migration of the bacteria. Therefore, the chemoattractant profile is assumed to be static throughout each simulation.

## Supplementary information

Supplementary Information

## Data Availability

The underlying data to produce the results for this article are available on Zenodo at https://doi.org/10.5281/zenodo.17392507.

## Code availability

The code to produce the results for this article are available on Zenodo at https://doi.org/10.5281/zenodo.17392507.
