# Supplementary material for: Study of wrap mode impact on Pseudomonas aeruginosa motion in the chemotactic field of fungi
Source: NPJ Syst Biol Appl. 2026 Jul 20;12:144. doi: 10.1038/s41540-026-00789-x (PMC13627651; doi:10.1038/s41540-026-00789-x)
Supplement: Supplementary file 1 [file 41540_2026_789_MOESM1_ESM.pdf]

Supplementary Information

Austin Hansen[1,2], Alireza Ramezani[2,3], Dale A. Pelletier[4], Tomás A. Rush[4],
Roya Zandi[2,3], Yi-Syuan Guo[4,5,6], Huijing Du[7], William Cannon[1,2,8], Mark Alber[1,2,9,*]

For calibration to *Pseudomonas aeruginosa* we use the experimental data found in [4]. However, we note that pull and push mode statistics for *P. aeruginosa* are very similar. Furthermore, other experimental data from *P. putida* suggests that pull mode takes up less than 2 percent of the total swimming time [1]. Thus, we model the push and pull mode as only one distinct mode which we refer to as "run" throughout this text.

Supplementary Note 1 – Model Calibration

1.1 Velocity

We fix a constant velocity for both run mode and wrap mode respectively. For both we choose the average velocity found for push and wrap mode as follows:

$$\text{Push Mode: } 37.95\frac{\mu m}{s}$$
$$\text{Wrap Mode: } 14.45\frac{\mu m}{s}.$$

This is done by adjusting the force $\boldsymbol{F}^i_{motor}$. Given the equation of motion,

$$\frac{d\boldsymbol{x}_i(t)}{dt} = -\frac{1}{\gamma}\nabla_x U(\boldsymbol{x}_i(t)), \tag{1}$$

we first note that $\boldsymbol{F}^i_{bond}$ and $\boldsymbol{F}^i_{bend}$ are internal forces which not contribute to the velocity of the bacteria. Hence, we have that

$$\begin{aligned}\frac{d\boldsymbol{x}_i(t)}{dt} &= -\frac{1}{\gamma}\nabla_x U(\boldsymbol{x}_i(t)) = \frac{1}{\gamma}\boldsymbol{F}(\boldsymbol{x}_i(t))\\ &= \frac{1}{\gamma}\boldsymbol{F}^i_{motor}.\end{aligned}$$

We define $\gamma = .01$. Thus, we have in run mode:

$$\begin{aligned}\frac{\boldsymbol{F}^i_{motor}}{.01} &= \frac{d\boldsymbol{x}_i(t)}{dt} = 37.95\\ \boldsymbol{F}^i_{motor} &= .3795.\end{aligned}$$

Similarly, in wrap mode we have,

$$\begin{aligned}\frac{\boldsymbol{F}^i_{motor}}{.01} &= \frac{d\boldsymbol{x}_i(t)}{dt} = 14.45\\ \boldsymbol{F}^i_{motor} &= .1445.\end{aligned}$$

1.2 Durations

We fit the durations of both wrap and run modes in the following manner. First, the experimental histograms in Figure 5A and Figure S6D in [4] were extracted using the function GRABIT in MATLAB. Then both μ and σ are found such that the error,

$$\sum_{i=1}^{N_{bins}} \left(f(x_i, \mu, \sigma) \frac{\text{max duration}}{\text{N}_{bins}} - y_i \right)^2, \tag{2}$$

is minimized. Here, f is the probability density function (pdf) for a truncated lognormal distribution, max duration is the maximum mode duration observed from experiments, and y_i is the experimental pdf for a given duration x_i. For run durations, we choose to calibrate to the push durations found in Figure S6D in [4] and exclude the first 2 bins from the calibration to get a more accurate fit. The following figures show both the extracted experimental data and the fit distribution. For run durations, we found the best fit parameters were $\mu = .7144$ and $\sigma = .7440$. For wrap durations, the best fit parameters were $\mu = -.3662$ and $\sigma = .91784$.

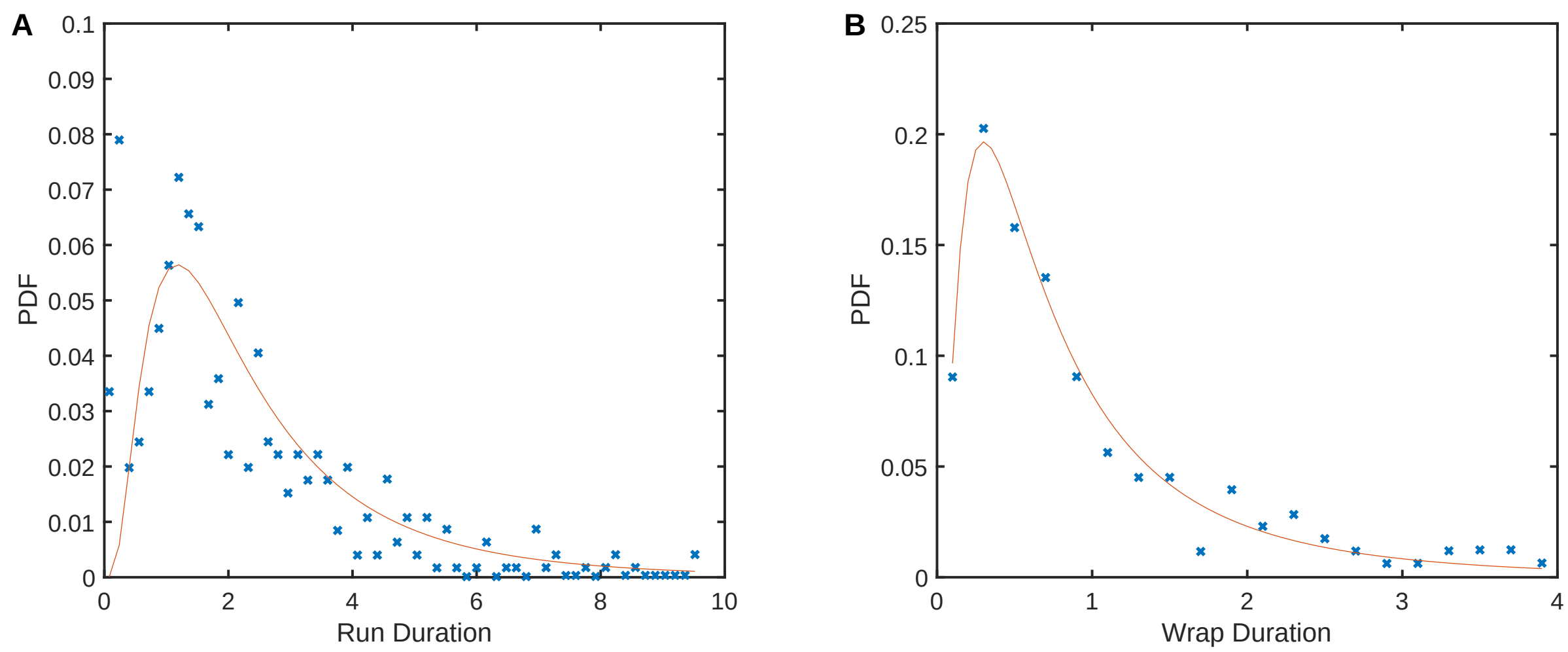

Supplementary Figure 1: Calibrated pdfs for both run durations (A) and wrap durations (B). The blue shows the experimental data, while the red shows the pdf of the truncated log-normal distribution.

1.3 Angular Changes

Finally, to calibrate both the angle change in reversal events and the angle change during wrap mode, we refer to Figure 5D and Figure 5C in [4], respectively.

Reversals

In our model, since the time during a reversal event is constant, the resulting angle is entirely determined by the angle of rotation in the force on the frontier node $\boldsymbol{F}^0_{motor}$ at each time step. In order to calibrate the angle of rotation R needed to match the experimental distribution, we first plot the relationship between R

and the total angle change during the reversal event. Furthermore, we limit R to only those values which produce angle changes between $90° - 180°$.

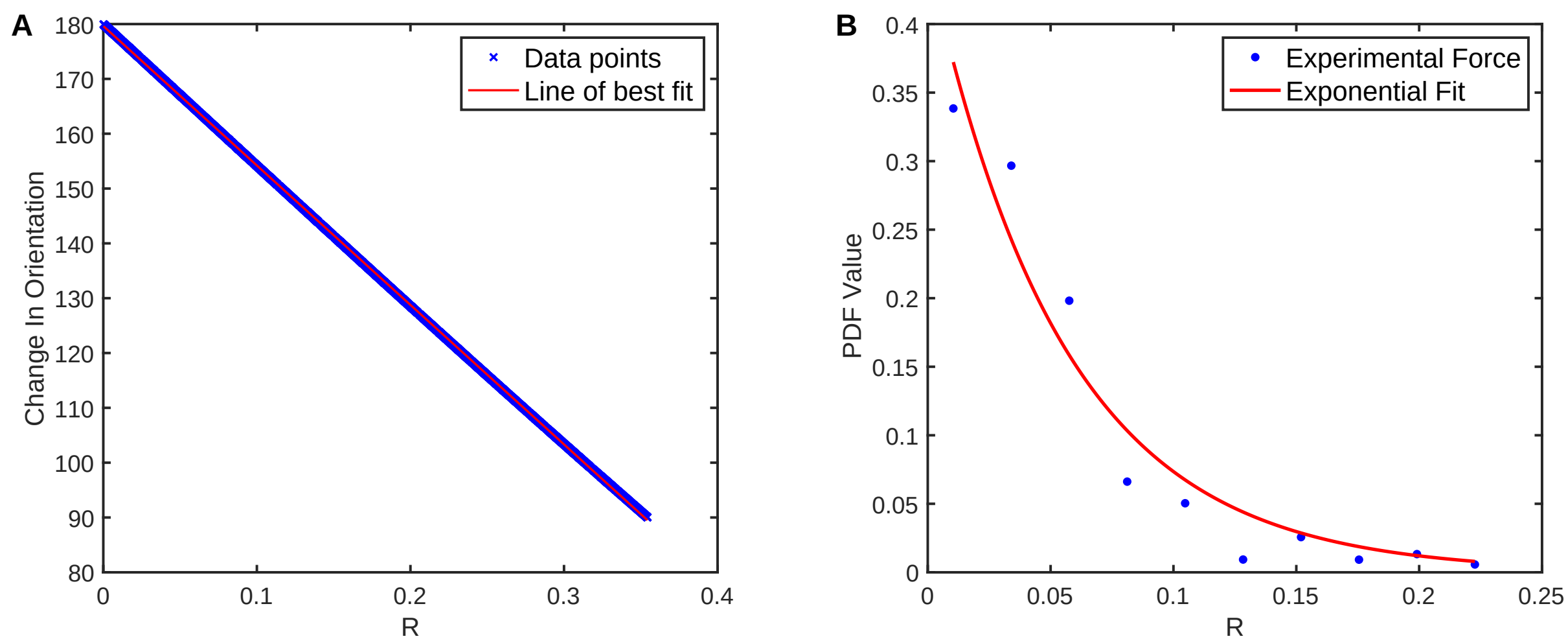

Supplementary Figure 2: (A) Relationship between the total angle change during reversal events and the angle of rotation in the force on the frontier node $\boldsymbol{F}^0_{motor}$ from our model simulations. (B) The relationship between the experimental pdf and the angle of rotation R. The blue dots show the experimental data and the red line shows the line of best fit.

Line of best fit for Figure 2A is

$$y = -254.40R + 179.65 \tag{3}$$

where y is the total change in orientation.

Given this relationship, in order to limit our angular changes between $90° - 180°$, we solve for the value of R that leads to a total angular change of $90°$

$$\begin{aligned} 90 &= -254.40R + 179.65 \\ -89.65 &= -254.40R \\ R &= \frac{89.65}{254.40} \end{aligned}$$

In contrast, when $R = 0$, the total angular change is 180°, since we reverse the order of nodes and $\boldsymbol{F}^0_{motor}$ is pointed along the orientation of the bacterium.

Now, using the linear function found above, we plot the experimental pdf, which can be made a function of R. Figure 2B shows this along with the line of best fit.

Here, the data was fit to an exponential, and the equation found is

$$E = 0.4495e^{-18.11R} \tag{4}$$

Finally, in order to convert this into a usable distribution, we create a cumulative distribution function (CDF) from this function using the fact that all possible angles of rotation should be between 0 and $\frac{89.65}{254.40}$.

To find the CDF, we first solve the following equation for a

$$1 = \int_0^{\frac{89.65}{254.40}} \frac{0.4495}{a} e^{-18.11R} \tag{5}$$

$$a \approx 0.0247785 \tag{6}$$

Then, the desired CDF is

$$F = \int_0^R \frac{0.4495}{.0247785} e^{-18.11R} dR = 1.0017 - 1.0017e^{-18.11R} \tag{7}$$

while the inverse of this is

$$R = \frac{\ln(\frac{F-1.0017}{-1.0017})}{-18.11} \tag{8}$$

Then, we can use inverse transform sampling to generate an angle of rotation sampled from the fit distribution. Now that we have the inverse CDF, F^{-1}, we can generate a random sample from a uniform distribution within [0,1] and then calculate the angle of rotation R such that $R = F^{-1}(u)$. This is then the angle of rotation R at each time step that is applied to the frontier force. Using this method, we plot the histogram from the calibrated parameters within our model. In Figure 3, we can see the histogram is a good fit to the experimental data.

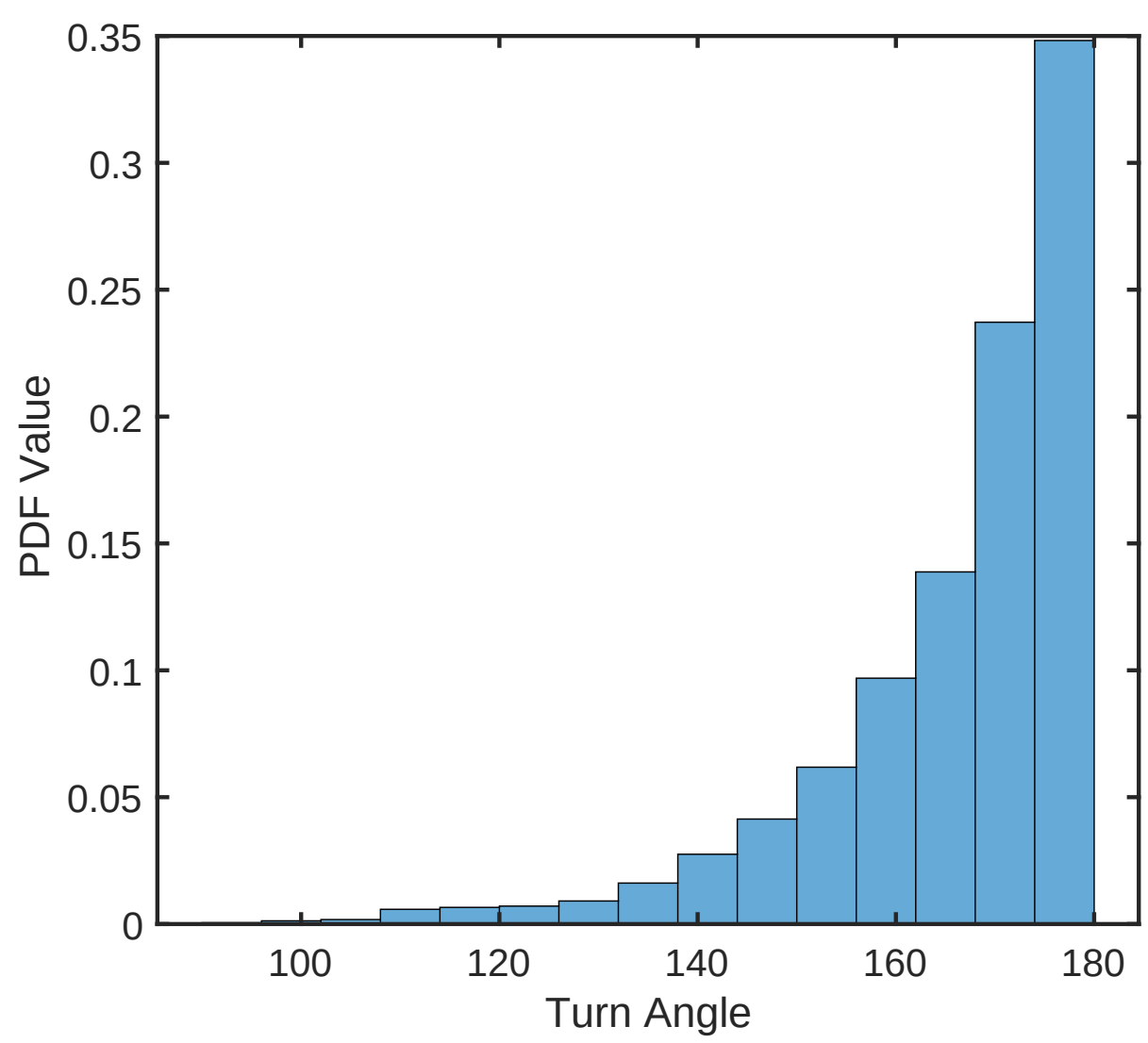

Supplementary Figure 3: Simulated histogram of the turn angles during reversal events with calibrated forces.

Wrap Mode

In order to calibrate the turn angle for wrap mode, we note that the angles presented in Figure 5A and 5E of [4] refer to the angle change made between pull and push modes. Thus, this includes both the angle change

from wrap mode itself as well as the change from a reversal event. Henceforth, we use the term "turn angle" to refer to the total angle change between the beginning of wrap mode and the end of the reversal event, while "wrap angle" will refer to the angle change only during wrap itself.

Data for Figure 5E of [4] was extracted and fit to an exponential function of the form $ae^{bx} + c$. Figure 4 shows both the extracted data and the exponential of best fit,

$$y = 51.08e^{-1.439d} + 87.02 \tag{9}$$

where d is the duration and y is the turn angle.

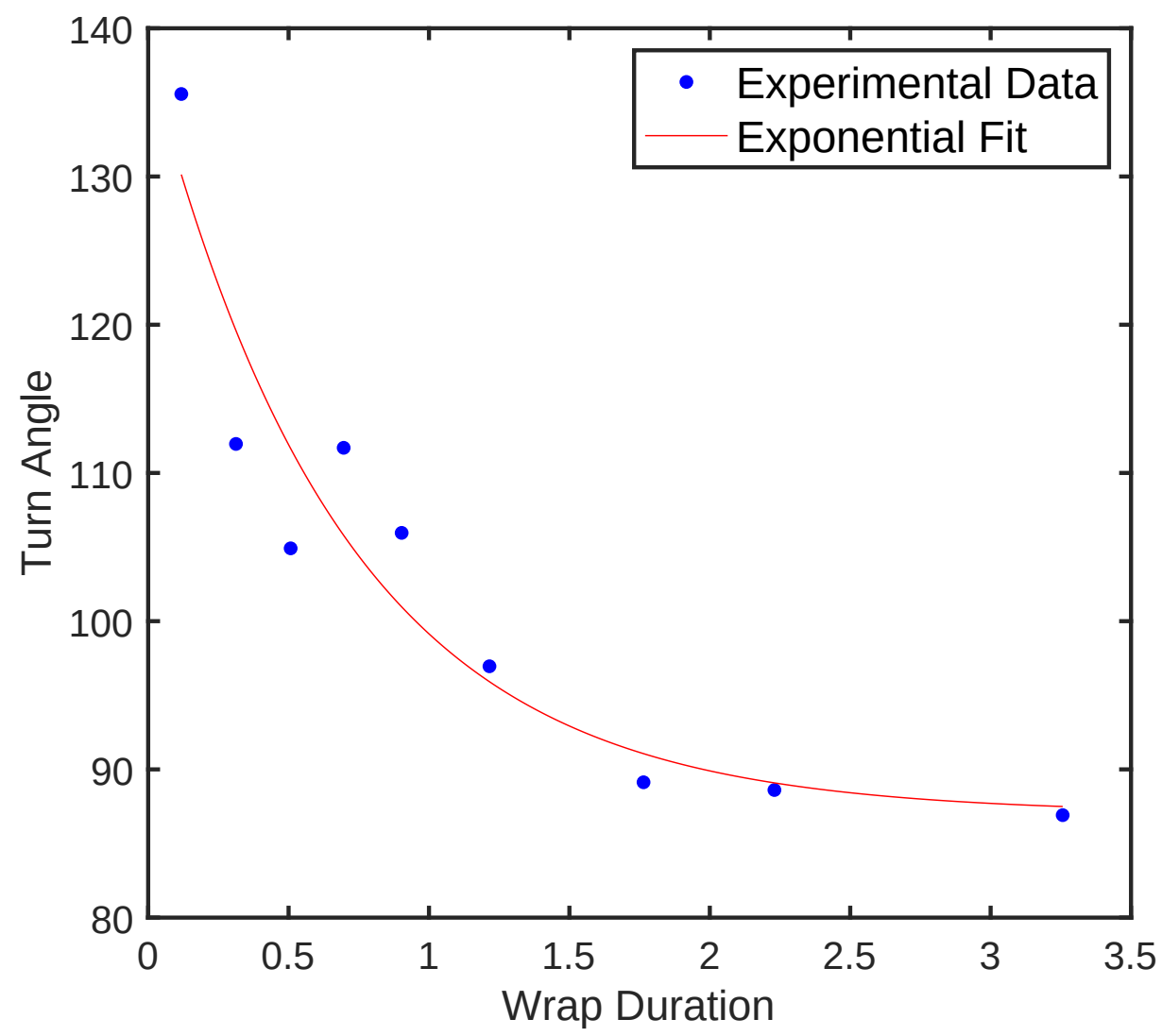

Supplementary Figure 4: Plot showing wrap duration vs turn angle. Blue is extracted from experimental figures and red is the line of best fit.

Since both duration and force affect the turn angle, we first sample a duration from the truncated log-normal distribution shown in Figure 1, and then use equation 9 to find the corresponding turn angle that that event should abide by. We note that reversals are, on average, centered around 180°. Furthermore, given that the turn angle is always less than 180° we have that TurnAngle = Reversal − WrapAngle. Hence, the wrap angle for this given turn angle is

$$W = 180 - (51.08e^{-1.439d} + 87.02) \tag{10}$$

Finally, we are left with finding the angle of rotation in the force on the frontier node $\boldsymbol{F}^0_{motor}$ that yields a wrap angle of W. This, however, can be done by noting that there is a linear relationship between R and wrap angle for a fixed duration. In order to deal with this relationship changing for each duration, we can normalize all the equations by dividing the wrap angle by the duration. For example, for durations of .5, 2,

and 3.5 seconds we have

$$\frac{W}{d} = 403.27 \cdot R + 0.03,$$
$$\frac{W}{d} = 397.12 \cdot R + 0.03,$$
$$\frac{W}{d} = 396.23 \cdot R + 0.03$$

respectively. Thus we approximate this relationship by the equation

$$\frac{W}{d} = 398.67 \cdot R \tag{11}$$

where R is the angle of rotation in the force on the frontier node $\boldsymbol{F}^0_{motor}$. This finally allows us to find the necessary value for R by solving for the equation

$$R = \frac{W}{398.67d} = \frac{180 - (51.08e^{-1.439d} + 87.02)}{398.67d} \tag{12}$$

For this calibration, we have the following calibrated figures resembling 5E and 5A of [4]:

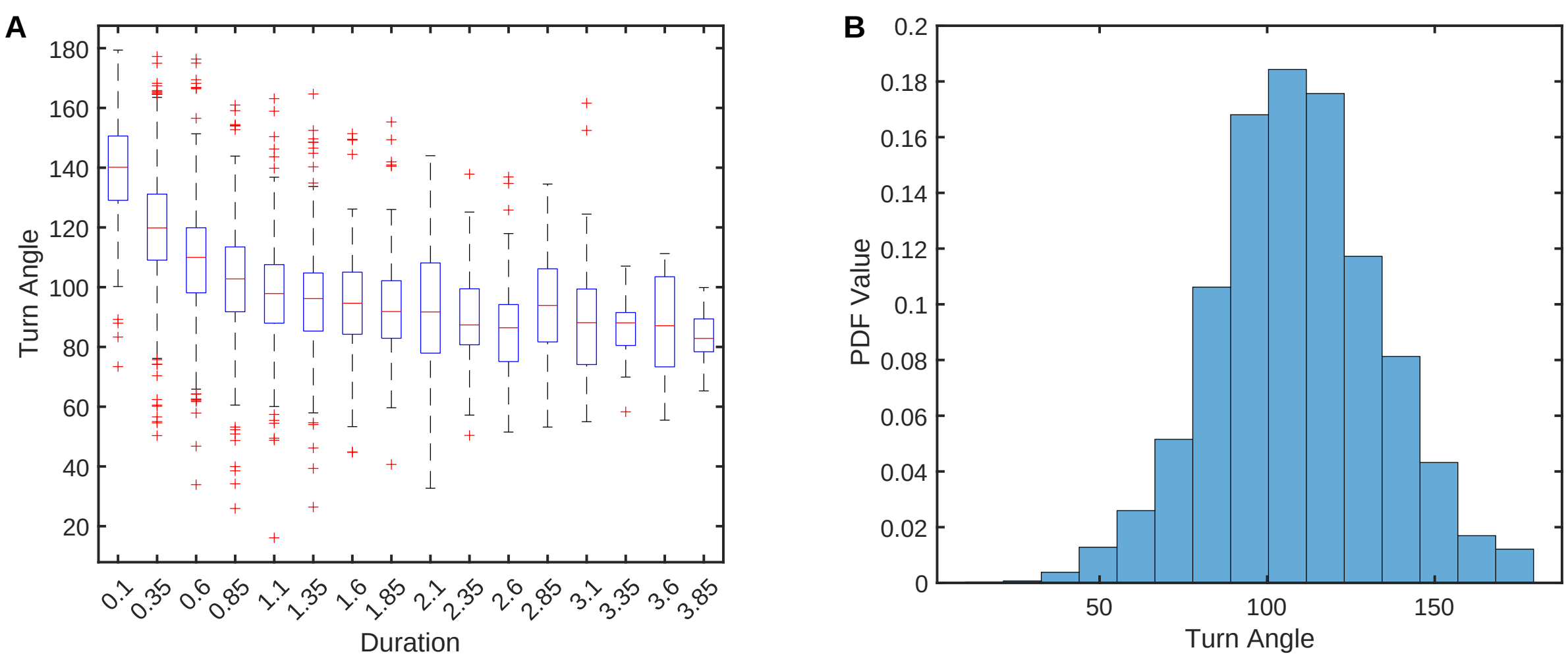

Supplementary Figure 5: (Left) shows boxplots of the turn angles during wrap mode for given durations. (Right) shows the pdf of the turn angles during wrap mode. Both figures were generated from simulated bacterial migration across 200s with no chemotaxis

Bending Coefficient

We note that in both reversal events and wrap mode the bending coefficient in the model allows each bacterium to bend slightly as the cell body undergoes changes in orientation. However, since the model was calibrated to specific angular distributions in wrap mode and reversal events, the exact calibration of

the bending coefficient is ultimately not impactful on the overall dynamics. The model results can easily be recovered at a variety of bending coefficients by first repeating the same calibration done in eq. 3. For example, for a bending coefficient of 1.0 we found that the relationship between R and the change in orientation of the bacterium was

$$y = -212.78R_{\text{new}} + 179.65 \tag{13}$$

Therefore we have,

$$R_{\text{new}} = \frac{-254.40}{-212.78}R_{\text{old}} \tag{14}$$

Thus, our final equation now becomes

$$R_{\text{new}} = \frac{-254.40}{-212.78}R_{\text{old}} = \frac{-254.40}{-212.78} \cdot \frac{\ln(\frac{F-1.0017}{-1.0017})}{-18.11} \tag{15}$$

In a similar manner for the wrap mode calibration we have

$$R_{\text{new}} = \frac{-254.40}{-212.78}R_{\text{old}} = \frac{-254.40}{-212.78} \cdot \frac{W}{398.67d} = \frac{-254.40}{-212.78} \cdot \frac{180 - (51.08e^{-1.439d} + 87.02)}{398.67d} \tag{16}$$

In figure 6 we show a portion of a trajectory using both a bending coefficient of 0.5 and 1.0 with and without the recalibrated forces. We see that we recover the same trajectory when the turn angles are properly calibrated to the experimental distributions.

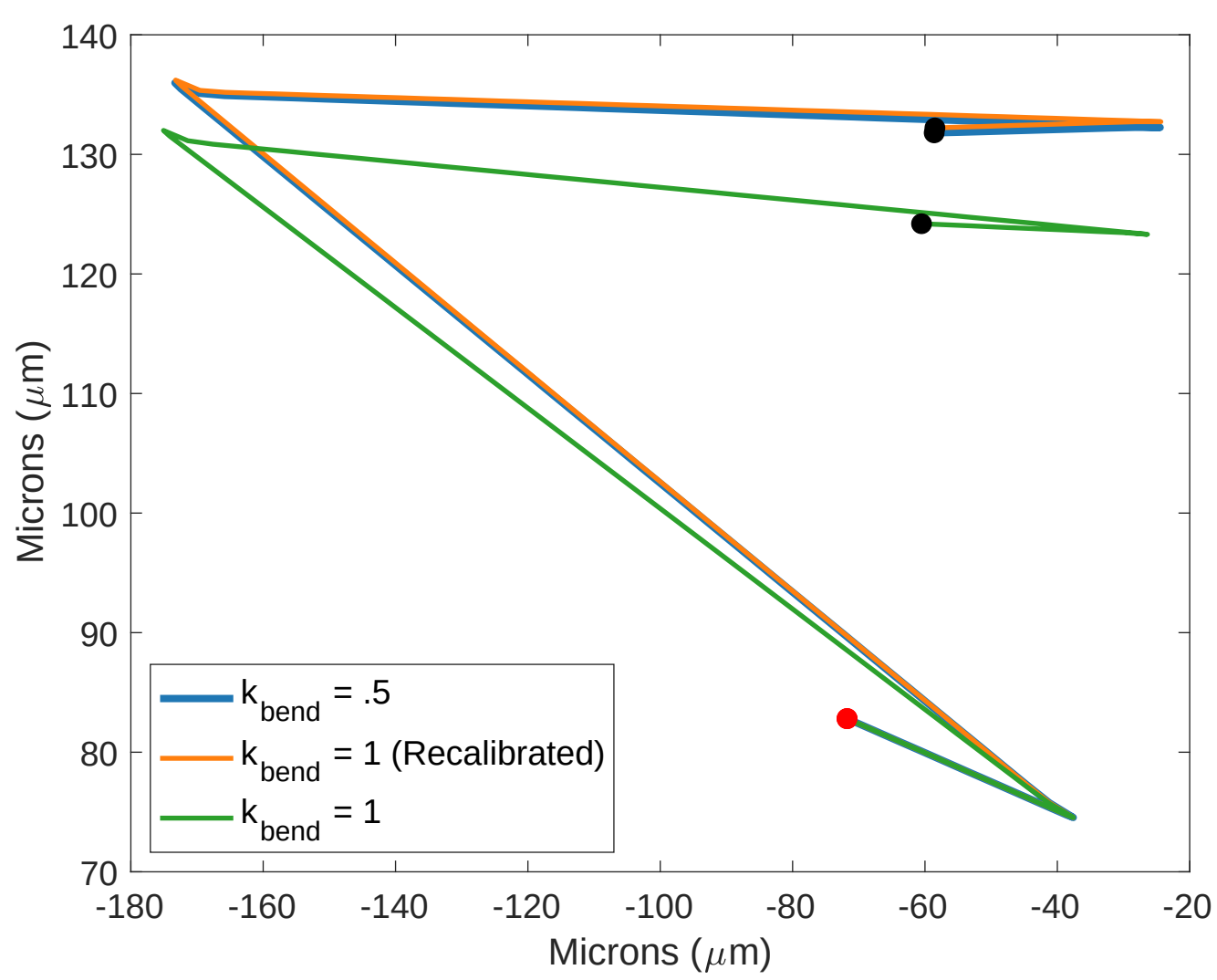

Supplementary Figure 6: A trajectory of a cell undergoing reversals with different values of k_{bend}. For the cell in blue and green, the only parameter in the model that is changed is k_{bend}, which is increased from 0.5 to 1.0. In orange we show the trajectory of a cell with a k_{bend} value of 1.0, however the angular changes have been calibrated to the experimental data. Here we see that we recover the same trajectory as that in blue.

Furthermore we note that the calibration used in this model is set to approximate a rigid rod. Following the strategy in [2] we utilize the relationship between the bending modulus B and the bending coefficient

k_{bend} through the following equation

$$B = \frac{\pi}{4} E r^4 \tag{17}$$

This assumes that each cell is an isotropic elastic rod with Young's modulus E and radius r. The bending coefficient is then connected to the bending modulus through the force required to bend the elastic rod into a U-shape

$$F_m \approx \frac{B}{L^2} \tag{18}$$

where L is the length of the cell. Given a SCE of N nodes in this shape, we have $(N-2)$ angles which all sum to π. Thus we can write the equation as

$$F_m = \sum_{i}^{N-2} k_{bend} \frac{\theta - \theta_{eq}}{\frac{L}{N-1}} \tag{19}$$

Setting these expressions equal and solving for k_{bend} yields:

$$k_{bend} = \frac{B}{\pi(N-1)L} \tag{20}$$

We now approximate the coefficient k_{bend} by using values of E found for *Pseudomonas aeruginosa* using atomic force microscopy in [3]. Planktonic cells were measured to have a mean of 50.8 kPa, vs 218.7 kPa for aggregate regions. Utilizing these values in eq. 17 and plugging into eq. 20 with a radius of $.5\mu m$, $N = 5$ nodes, and a cell length of $L = 2$ μm yields $k_{bend} \in [0.099fJ, \quad 0.427fJ]$. Since the value for k_{bend} used in the model is several order of magnitudes larger than this the model presented here approximates a rigid rod.

Supplementary Note 2 – Chemoattractant Profile Generation

Here, we describe how the concentration for linear and exponential gradients are calculated.

2.1 Linear

For linear gradients, we use the following analytical expression to generate gradients with different slopes. C is the concentration of the chemoattractant and is calculated by

$$c_{i,j} = a\left(\sqrt{\left(\frac{domainX}{2}\right)^2 + \left(\frac{domainY}{2}\right)^2} - \sqrt{(i - cntrX)^2 + (j - cntrY)^2}\right), \tag{21}$$

where (i, j) is a grid point in the domain and $(cntrX, cntrY)$ is the center of the domain. This produces a linear gradient with slope a at every point. Figure 7 shows the profile and a plot of the concentrations along the 1-dimensional diagonal cross section.

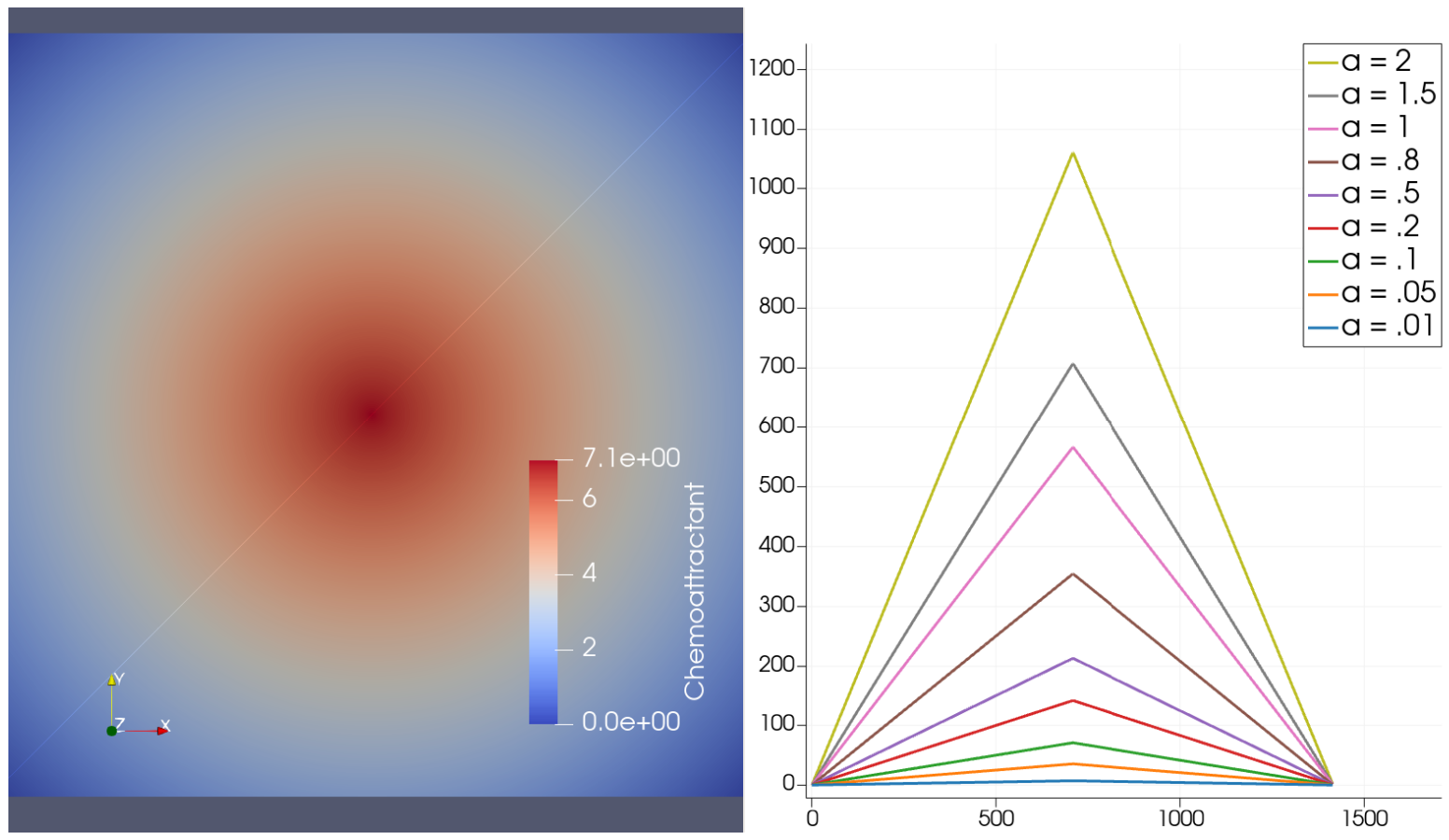

Supplementary Figure 7: Chemoattractant profile with various linear slopes, a. Right shows a the profile on a red-blue scale where red indicates the highest concentrations and blue represents the lowest. Left shows the concentration plotted across the diagonal cross section for different values of a.

2.2 Exponential

In order to generate an exponential profile, we use a forward Euler method to solve a reaction-diffusion equation for an arbitrary chemoattractant. The domain is discretized into squares with length 5 μm, and the chemoattractant can diffuse from one square into the 4 neighboring squares. We assume that the time scale of diffusion for the chemoattractant is faster than that of the bacteria. Therefore, we calculate the profile before initializing the bacteria colony. The reaction-diffusion equation is solved with no-flux boundary conditions and is run for 10,000 steps. The resultant profile is then interpolated to a 1 μm grid and taken to be the static profile used throughout the whole simulation. In the following, we show the resultant profiles for various production rates when the diffusion coefficient is set to 200.

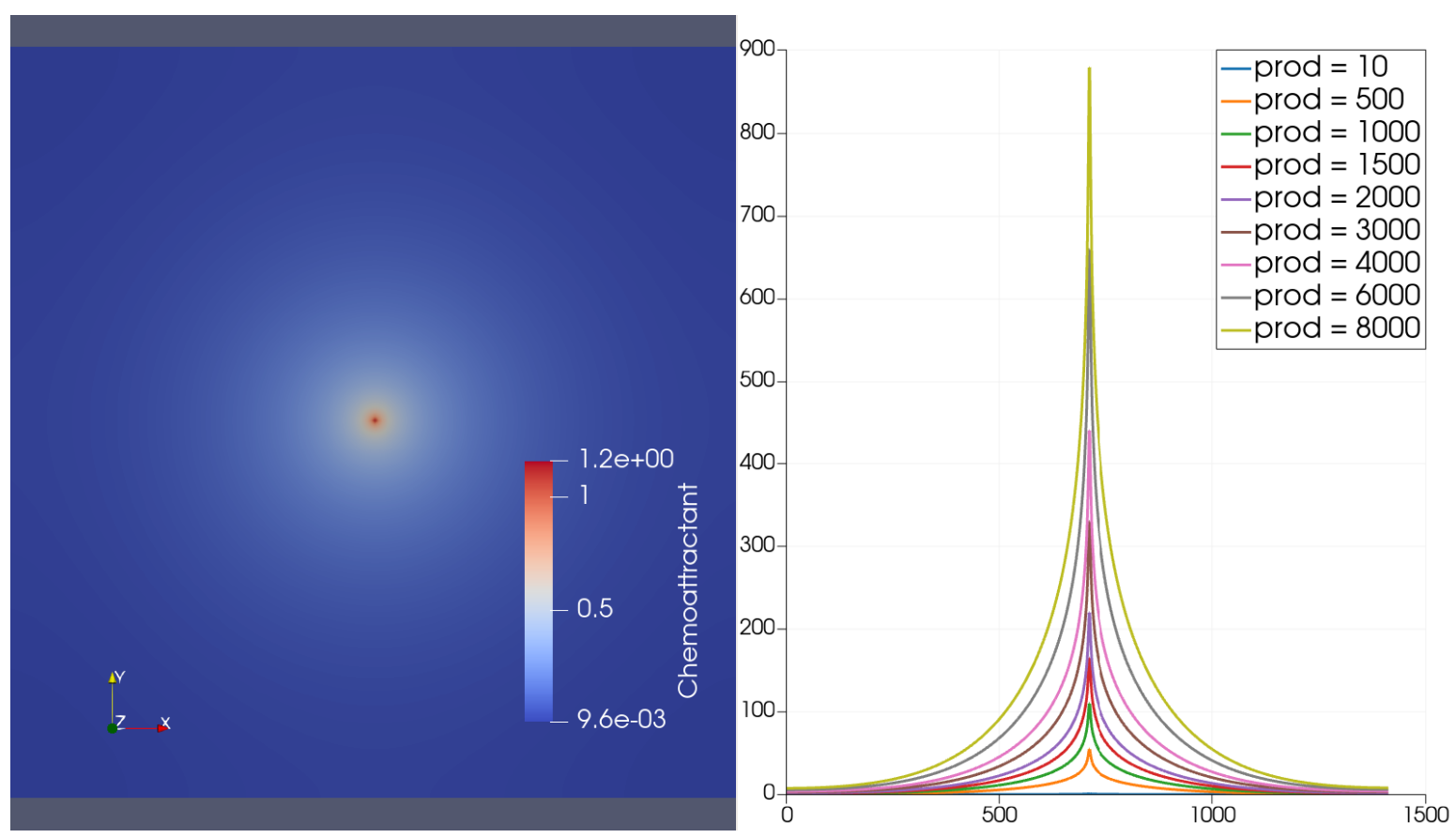

Supplementary Figure 8: Chemoattractant profile with various production rates. Right shows a the profile on a red-blue scale where red indicates the highest concentrations and blue represents the lowest. Left shows the concentration plotted across the diagonal cross section for different productions values. All the concentrations are generated using a reaction-diffusion equation.

Supplementary Note 3 – Model Parameters

Supplementary Table 1: Model Parameters

Parameter	Value	Units	Description
N_b	*	-	Number of Bacteria
$L_{bacteria}$	2.0	μm	Length of bacteria
N_{nodes}	5.0	-	Number of nodes in each bacteria
l_{eq}	0.4	μm	Equilibrium length of linear spring
P_{wrap}	*	-	Probability of Wrap Mode
k_b	5.0	$\mu N \cdot \mu m^{-1}$	Stiffness of linear spring
k_{bend}	0.5	$\mu N \cdot \mu m \cdot rad^{-1}$	stiffness of torsional spring
θ_{eq}	π	rad	Equilibrium angle of torsional spring
γ	0.01	$\mu N \cdot s \cdot \mu m^{-1}$	Damping coefficient
$f_{motor,run}$	.3795	μN	Motor force on each node in run mode
$f_{motor,wrap}$	.1445	μN	Motor force on each node in wrap mode
μ_{run}	.7144	s	Lognormal-Truncated Mean for run mode duration
$\sigma_{t,run}$	.7440	s	Lognormal-Truncated SD for run mode duration
$[a_1, b_1]$	$[0, 9.6]$	s	Range of durations for run mode
μ_{wrap}	$-.3662$	s	Lognormal-Truncated Mean for wrap mode duration
$\sigma_{t,wrap}$	.91784	s	Lognormal-Truncated SD for wrap mode duration
$[a_2, b_2]$	$[0, 4]$	s	Range of durations for wrap mode
t_{turn}	.2	s	Duration of reversal events
ζ	*	-	Chemotaxis sensitivity
$\triangle t_\zeta$	0.1	s	Time between updates to chemotactic model
$L_x \times L_y$	*	-	Domain size
R_0	*	μm	Initial distance from the source
dt	0.00001	s	Simulation time step
T_{total}	*	s	Total simulation time
**Reaction Diffusion Parameters**			
D	200	$\frac{\mu m^2}{a.u\, of\, time}$	Diffusion coefficient
p	1000	$a.u^{-1}$	Production rate of attractant
dt_{chem}	.05	$a.u\, of\, time$	Chemical model's time step
$\triangle x \times \triangle y$	5×5	μm^2	Chemical Grid Step Size
t_{chem}	10000	$a.u\, of\, time$	Total duration of reaction diffusion model

* Parameters varied in simulation runs

Supplementary Note 4 – Chemotactic Model Parameter Exploration

This section explores the relationship between the chemosensitivity parameter, the gradient, and bacterial aggregation. We show that the equilibrium solution for bacteria in a linear gradient is dependent on the strength of the gradient, while varying exponential gradients produce the same equilibrium solution given enough time.

4.1 Increase in chemosensitivity results in greater chemotactic efficiency.

We first examine bacterial migration using a run-reverse pattern with different bacterial sensitivities ζ to the chemoattractant. These simulations show that aggregation towards a point source increases with the sensitivity, ζ. We use three metrics for quantifying the chemotactic efficiency of the bacteria: the average distance to the source $\bar{D}$, drift velocity defined as,

$$v_d = \frac{Starting\ Distance - Distance\ To\ Source}{\Delta t}, \tag{22}$$

and aggregation time τ_{ag}, which is defined to be the average time a bacteria spends within a given radius of the source. The radius here was chosen to be 50 μm. Figure 9. shows the simulation results for a fixed chemoattractant profile across sensitivity levels $\zeta = 1, 2, 5, 10, 20, 50$. The chemoattractant profile was generated using a reaction-diffusion equation with the parameters listed in table 1. Each simulation was run with 200 cells, initialized in a circle around a point source. The total domain size was $1000\,\mu m \times 1000\,\mu m$.

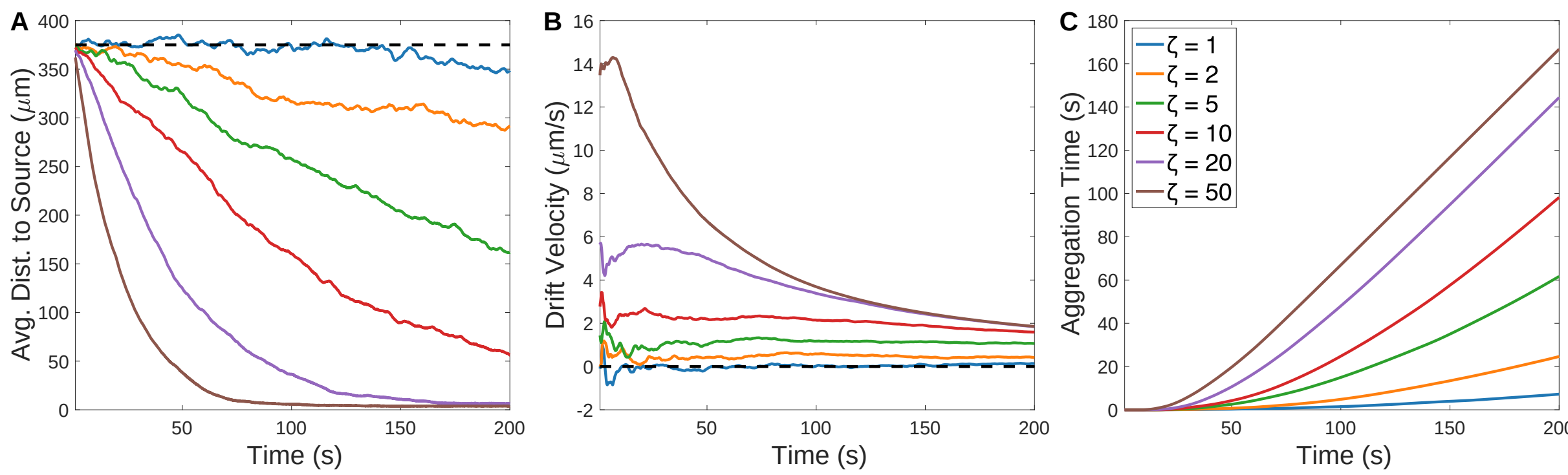

Supplementary Figure 9: Simulations showing bacterial migration with a run-reverse pattern in the presence of a point source and varying chemo-sensitivities: (A) average distance to the source, (B) drift velocity, and (C) aggregation time as a function of time.

Fig. 9A shows that as the sensitivity parameter ζ increases, the average distance to the source $\bar{D}$ decreases. This demonstrates that as the bacteria become more sensitive to the chemoattractant gradient and modulate their reversal frequencies more aggressively, they are able to find the source faster. We see that when $\zeta = 1$, the overall decrease in average distance is very small, however, for the largest sensitivity tested, $\zeta = 50$, the average distance decreases to below $50\,\mu m$ by around $45\,s$ in the simulation. In Figure 9B we see that the drift velocity increases as the sensitivity increases. For $\zeta = 1$ we have a max drift velocity of 0.96 $\frac{\mu m}{s}$, however, for $\zeta = 50$ there is a maximum drift velocity of 14.29 $\frac{\mu m}{s}$. Since drift velocity measures how much migration towards the source has occurred relative to simulated time, we see that for larger sensitivities, there is a spike at the beginning of the simulation and a gradual decrease as the simulation goes on. This is because the bacteria travel as close to the source as possible very early in the simulation and then remain there for the rest of the simulated time. This is further reflected in the metric τ_{ag}. Here we see that for $\zeta = 1$, the bacteria spend on average $7\,s$ in the center $50\,\mu m$ of the domain. However, for $\zeta = 50$, this increases to an average of $166.98\,s$. This shows that with larger sensitives, the bacteria are not only able to find the source quicker, but they remain here throughout the rest of the simulated time.

4.2 Chemoattractant gradient determines the average distance to the source at equilibrium

Next, we examine how varying the chemoattractant gradient between a linear and exponential profile impacts the chemotactic efficiency of bacteria with a fixed chemoattractant sensitivity. Simulations are run on the same domain size and initial conditions as section 4.1, except the total simulation time is 1600 seconds. The chemosensitivity is set to 1 and 200 cells are simulated for each simulation.

Linear Gradient

We turn our attention first to the simulated results of bacterial migration in the presence of a linear gradient. In Figure 10 we can see that as we increase the slope of the gradient, we have a decrease in the average distance to the center $\bar{D}$, and an increase in drift velocity v_d and aggregation time τ_{ag}. We note, however,

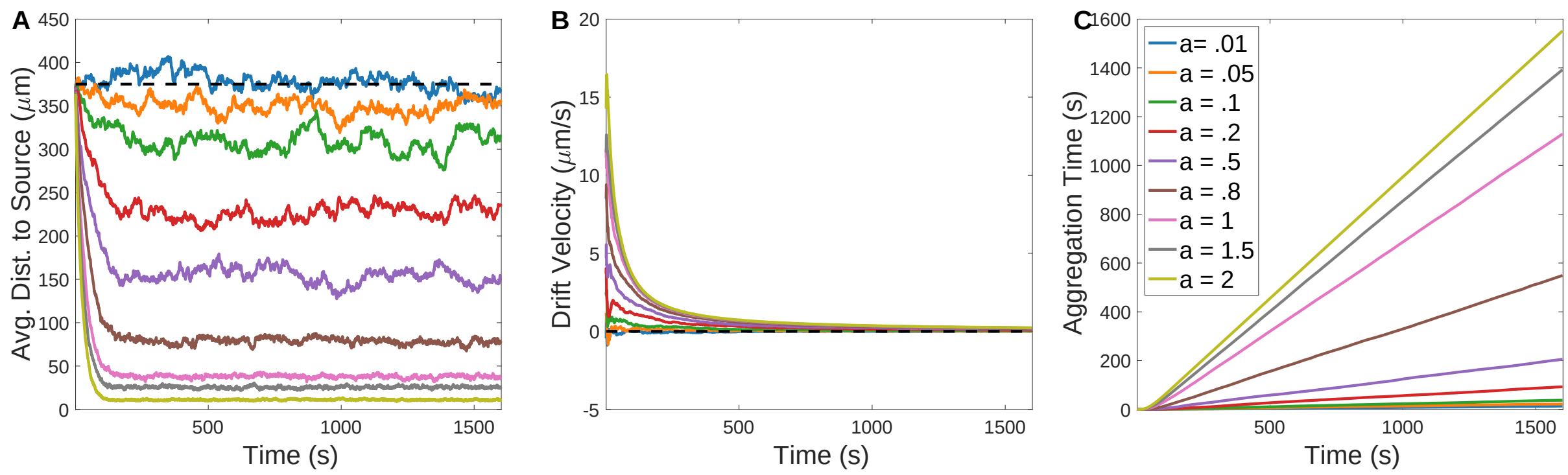

Supplementary Figure 10: Simulations showing bacterial migration with a run-reverse pattern in the presence of a linear gradient for various slopes. A) shows the average distance to the source, B) shows the drift velocity, and C) shows the aggregation time.

that varying the strength of the gradient seems to yield a different equilibrium solution. For example, given the largest gradient ($a = 2$), we see that $\bar{D}$ decreases rapidly until around $95\,s$. The bacteria then roughly remain around $10\,\mu m$ for the rest of the simulation. This property can be seen across all the different gradients. For example, for $a = .5$ we see that after the initial descent, the bacteria remain mainly around $150\,\mu m$. This behavior suggests that the value of the equilibrium solution is directly related to the strength of the gradient. Furthermore, we note that the variance in the fluctuations around each equilibrium solution is inversely related to the strength of the gradient, with the fluctuations being reduced as the gradient increases. Finally, we see that if the gradient is too small, ($a < .01$), the bacteria have a hard time moving towards the source and are largely indistinguishable from purely random motion.

Exponential Gradient

In Figure 11 we look at how *Pseudomonas aeruginosa* responds to the presence of an exponential gradient. Here we see a much different property. For low production rates ($prod = 10$, $prod = 500$) the migration is relatively random. However, as we start to increase the production rate, we see that $\bar{D}$ steadily trends downward throughout the whole simulation. In the case of the highest tested production rates, $\bar{D}$ only levels off when it has decreased below $50\,\mu m$. This is likely because near the starting location the gradients

are very weak, yet near the center the gradients become increasingly large. This difference causes the cells to move nearly randomly in the low-gradient environment until they find a region of the domain with a sufficiently high gradient to reliably guide the cells to the source. The smaller the source term, the smaller this region will be compared to the whole domain. However, once a cell finds the source, it does not leave this region since the exponential gradients here have become so large that they reverse almost immediately when moving down the gradient.

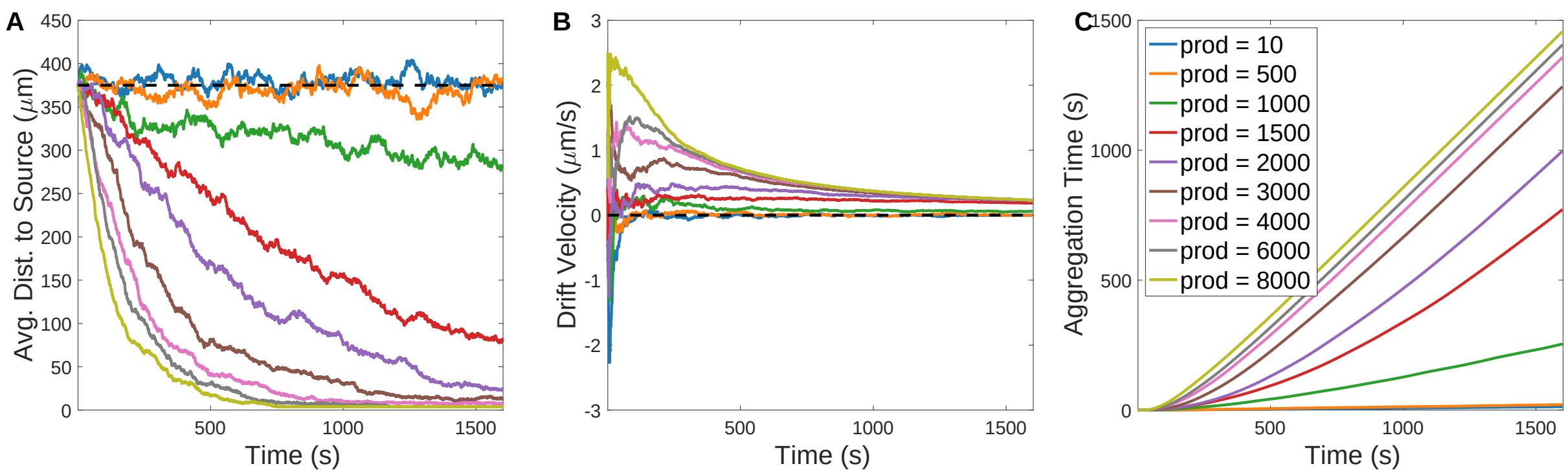

Supplementary Figure 11: Simulations showing bacterial migration with a run-reverse pattern in the presence of an exponential gradient for various production rates. A) shows the average distance to the source, B) shows the drift velocity, and C) shows the aggregation time.

Supplementary Note 5 – Model of Fungal Network

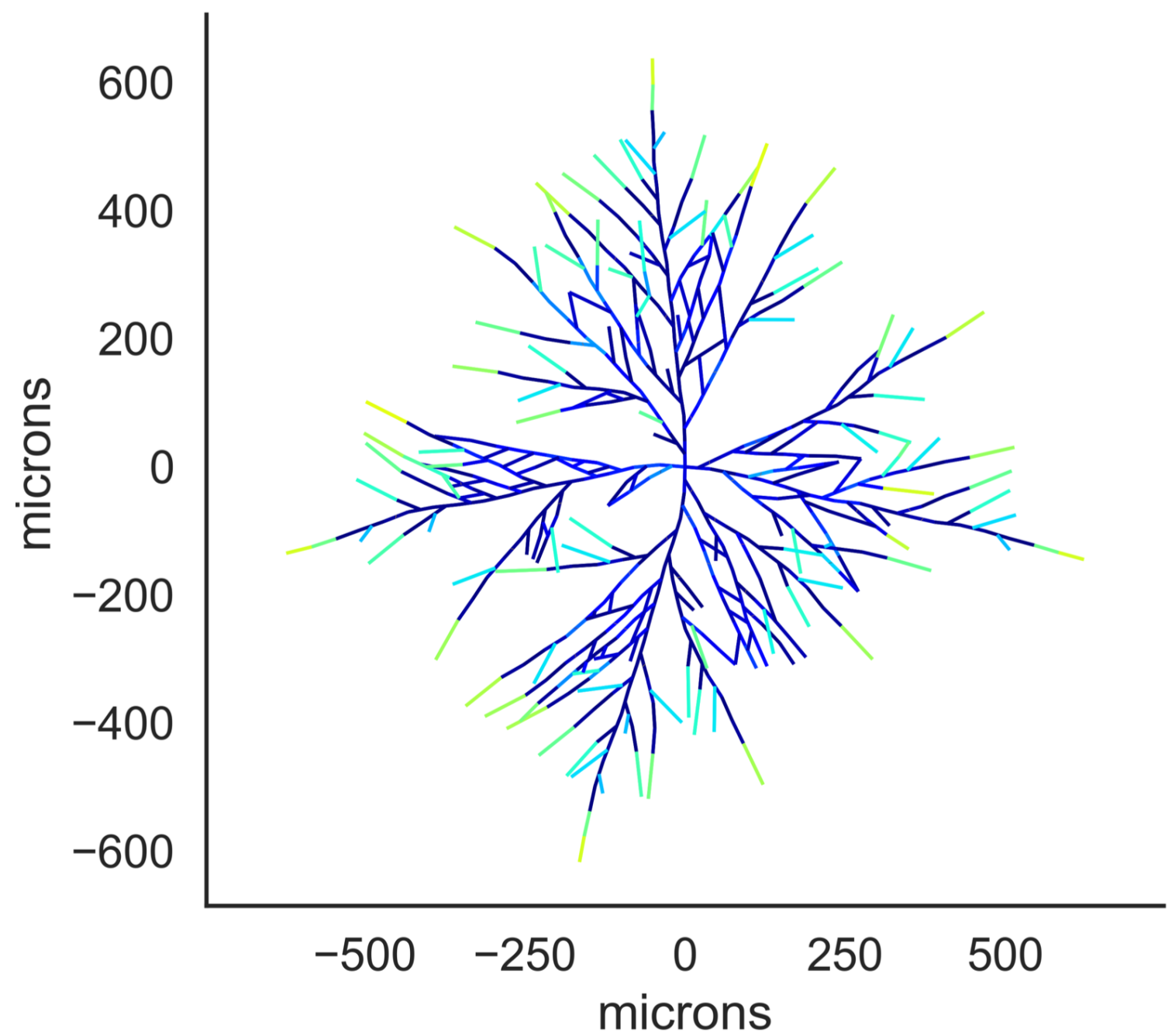

Supplementary Figure 12: Model hyphal structure. The hyphal length of 40 microns is comparable to many species of *Penicillium*.

Supplementary Note 6 – Wrap Mode Benefit is Dependent on Strength of Gradient

The following section outlines how the benefits of wrap mode only occur within a certain range of gradients. Since bacteria are made more responsive by increasing the chemosensitivity parameter as well as increasing the gradient, this result can also be framed by saying that wrap mode only shows benefits within a set range of chemotactic sensitivities. For both smaller and larger gradients, we see that 0% and 38% probability of wrap mode show very similar results. This is because wrap mode increases the diffusion coefficient in low-gradient environments. If the gradient is not large enough to meaningfully impact the run and wrap durations, then this increased diffusion does not correspond to an increased aggregational efficiency. Conversely, if the gradient is very large, cells in run mode reverse quickly when moving down the gradient. If this occurs quickly enough, then any increased angular exploration from wrap mode is able to be achieved by repeated reversal events.

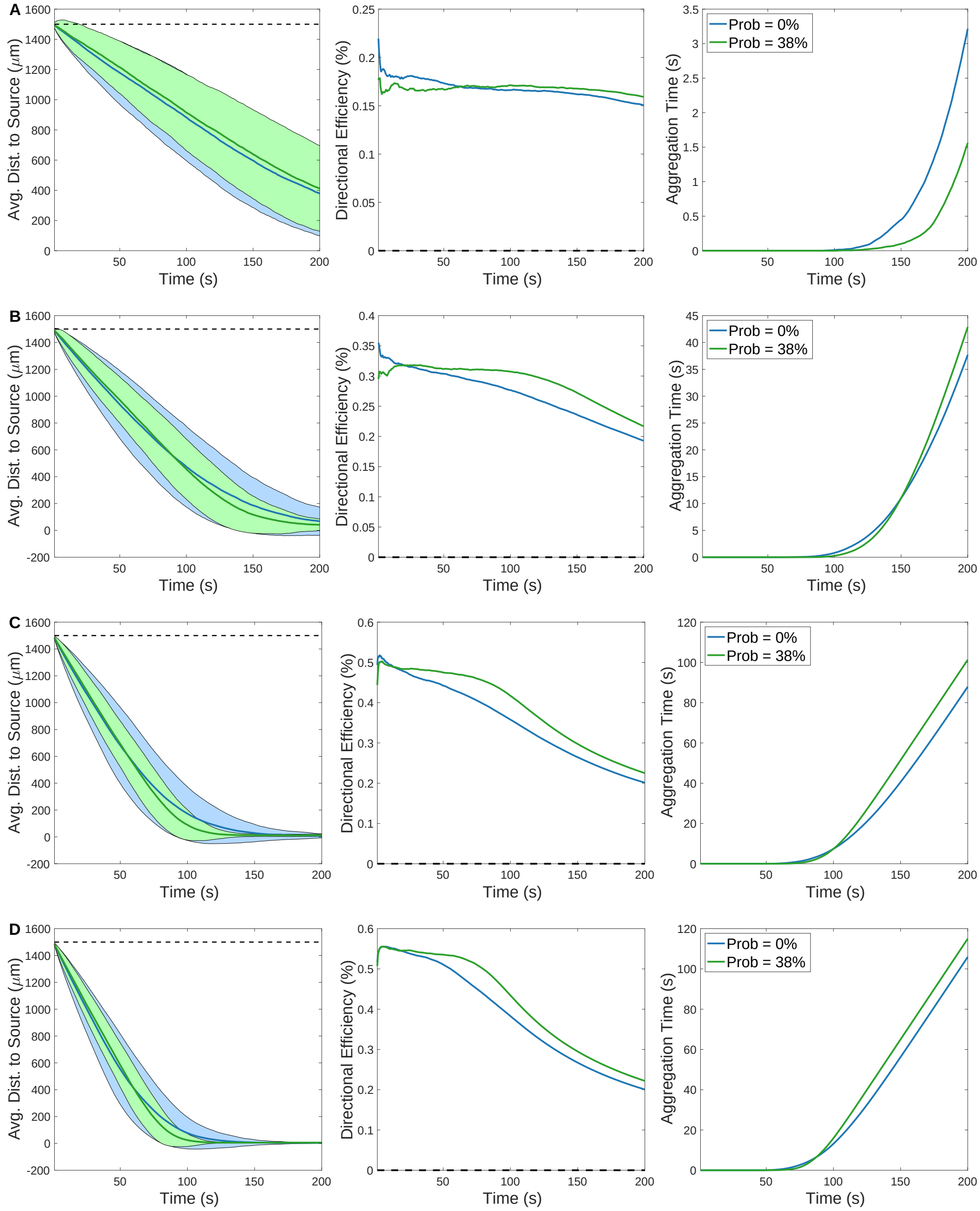

Supplementary Figure 13: Bacterial migration in response to different linear gradient profiles (A) Slope of $\frac{1}{2}$ (B) Slope of 1 (C) Slope of 2 (D) Slope of 4. We see that wrap mode gives the most benefit for intermediate chemoattractant gradients.

Supplementary Note 7 – Wrap Mode Benefit is Influenced by Level of Noise During Run Mode

In addition to the strength of the gradient, we also test whether the level of noise in the motion of the bacteria during run mode effects the distributions of a run-reverse strategy and a run-wrap-reverse strategy. In the simulations presented above each bacterium swims in a straight line during run mode. While this approximates behavior in perfectly homogeneous liquid environments, we now examine the effects of introducing noise into the run mode. This is modeled as a stochastic perturbation in the direction of the force applied to the frontier node. Thus, instead of $\boldsymbol{F}^0_{motor} = f_{motor}\hat{\boldsymbol{r}}_{0,1}$ we set $\boldsymbol{F}^0_{motor} = f_{motor}\hat{\boldsymbol{r}}^{\dagger}_{0,1}$, where $\hat{\boldsymbol{r}}^{\dagger}_{0,1}$ is a unit vector with angle $\bar{\theta}_{0,1} = \theta_{0,1} + \zeta \cdot D_r$, while $\theta_{0,1}$ is the angle between the vector $\boldsymbol{r}_{0,1}$ and the x-axis, and $\zeta \sim \mathcal{N}(0,1)$. We tested this both with updating $\hat{\boldsymbol{r}}^{\dagger}_{0,1}$ every time step and every hundred time steps at various levels of D_r. We found that the aggregation towards linear gradients were only effected when $\hat{\boldsymbol{r}}^{\dagger}_{0,1}$ was updated every hundred time steps and for higher values of D_r In Supplementary Figure 15 we show the distributions of wrap vs no wrap mode with $D_r = \{\sqrt{.002}, \sqrt{.02}, \sqrt{.2}\}$ updated every 100 time steps. Here we can see that Figures A-C look very similar to Figure 4A,B,C however when we increase the value of D_r, we see that the differences between the average distance to the source and aggregation time go away completely. Since noise increases the angular exploration of the cells, these results show that with sufficient noise in the run mode we achieve most of the same benefits of wrap mode. This supports the hypothesis that one of the key functions of wrap mode is to increase angular exploration. In Supplementary Figure 14 we also show a sample trajectory of a cell with $D_r = \sqrt{.2}$

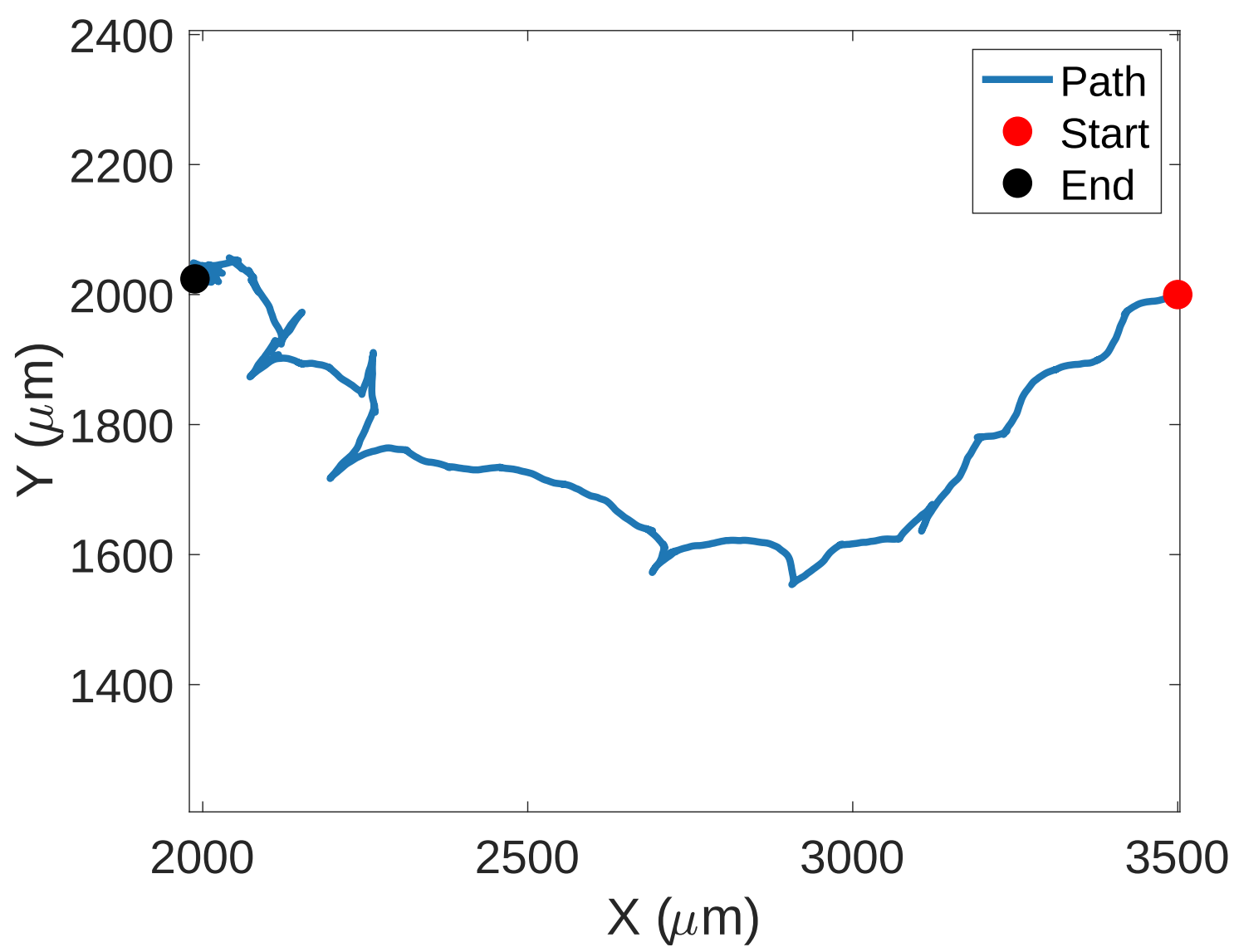

Supplementary Figure 14: The trajectory of a simulated cell during the first hundred seconds of the simulation. The simulation was ran with the same setup as in Figure 4A,B,C of the main text, except noise was introduced into the run mode. Here $D_r = \sqrt{.2}$

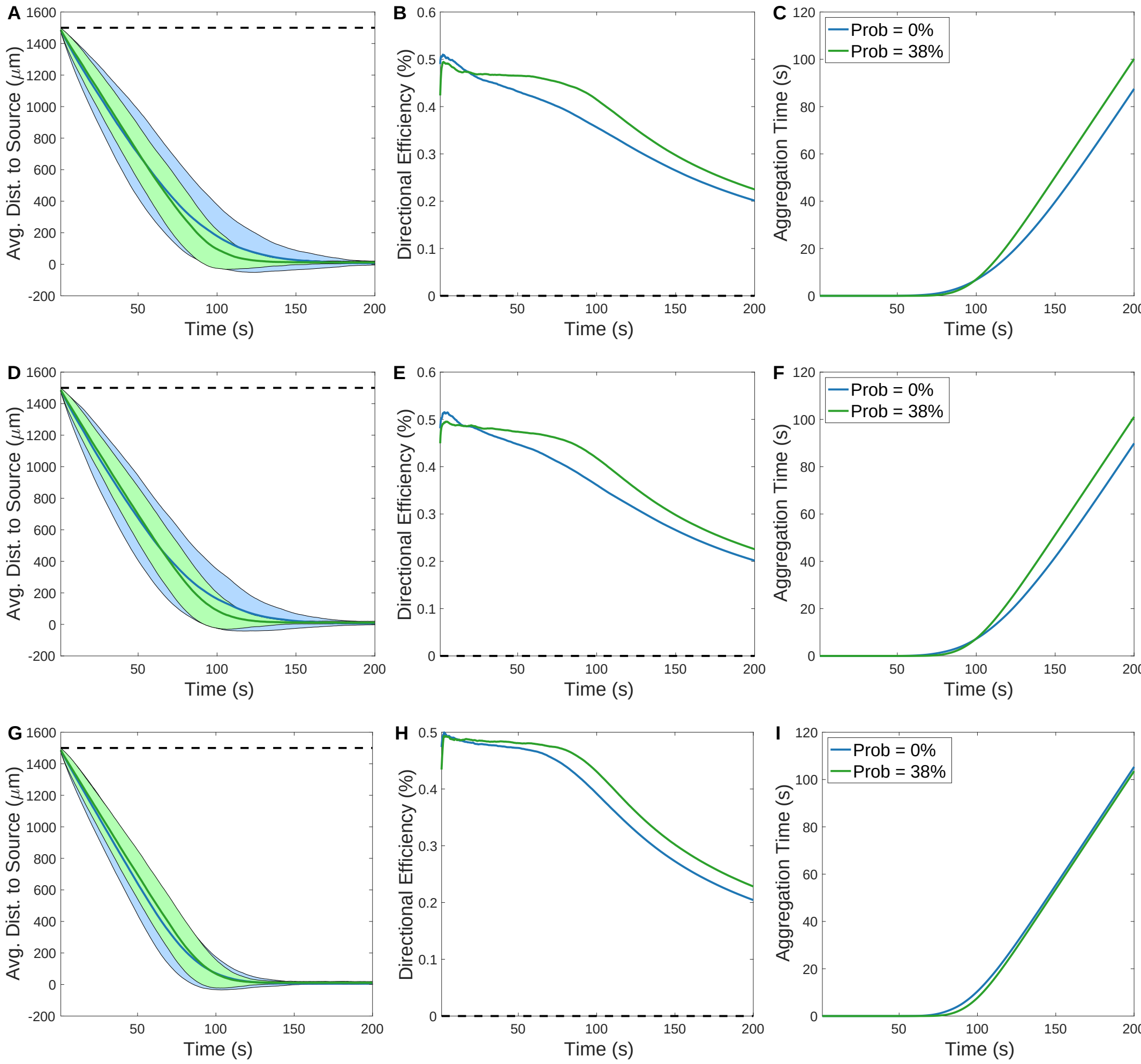

Supplementary Figure 15: Bacterial migration in response to a linear gradient with a slope of 2 but with different levels of noise in run mode. (A)$D_r = \sqrt{.002}$ (B)$D_r = \sqrt{.02}$ (C)$D_r = \sqrt{.2}$ We see that as we increase the level of noise the difference between wrap and cells without wrap mode is reduced.

References

[1] Zahra Alirezaeizanjani et al. "Chemotaxis strategies of bacteria with multiple run modes". eng. In: *Science Advances* 6.22 (May 2020), eaaz6153. ISSN: 2375-2548. DOI: 10.1126/sciadv.aaz6153.

[2] Cameron W Harvey et al. "Study of elastic collisions of *Myxococcus xanthus* in swarms". In: *Physical Biology* 8.2 (Apr. 2011), p. 026016. ISSN: 1478-3975. DOI: 10.1088/1478-3975/8/2/026016. URL: https://iopscience.iop.org/article/10.1088/1478-3975/8/2/026016 (visited on 02/28/2026).

[3] Caroline D. Miller, Meisam Asgari, and Sophie E. Darch. "AFM characterization of early *P. aeruginosa* aggregates highlights emergent mechanical properties". en. In: *mSystems* 10.11 (Nov. 2025). Ed. by Neha Garg, e01312–25. ISSN: 2379-5077. DOI: 10.1128/msystems.01312-25. URL: https://journals.asm.org/doi/10.1128/msystems.01312-25 (visited on 02/28/2026).

[4] Maojin Tian et al. "A new mode of swimming in singly flagellated Pseudomonas aeruginosa". eng. In: *Proceedings of the National Academy of Sciences of the United States of America* 119.14 (Apr. 2022), e2120508119. ISSN: 1091-6490. DOI: 10.1073/pnas.2120508119.
